# Efficient expression of full-length antibodies in the cytoplasm of engineered bacteria

**DOI:** 10.1038/ncomms9072

**Published:** 2015-08-27

**Authors:** Michael-Paul Robinson, Na Ke, Julie Lobstein, Cristen Peterson, Alana Szkodny, Thomas J. Mansell, Corinna Tuckey, Paul D. Riggs, Paul A. Colussi, Christopher J. Noren, Christopher H. Taron, Matthew P. DeLisa, Mehmet Berkmen

**Affiliations:** 1 School of Chemical and Biomolecular Engineering, Cornell University, Ithaca, New York 14853, USA; 2 New England Biolabs, 240 County Road, Ipswich, Massachusetts 01938, USA

## Abstract

Current methods for producing immunoglobulin G (IgG) antibodies in engineered cells often require refolding steps or secretion across one or more biological membranes. Here, we describe a robust expression platform for biosynthesis of full-length IgG antibodies in the *Escherichia coli* cytoplasm. Synthetic heavy and light chains, both lacking canonical export signals, are expressed in specially engineered *E. coli* strains that permit formation of stable disulfide bonds within the cytoplasm. IgGs with clinically relevant antigen- and effector-binding activities are readily produced in the *E. coli* cytoplasm by grafting antigen-specific variable heavy and light domains into a cytoplasmically stable framework and remodelling the fragment crystallizable domain with amino-acid substitutions that promote binding to Fcγ receptors. The resulting cytoplasmic IgGs—named ‘cyclonals'—effectively bypass the potentially rate-limiting steps of membrane translocation and glycosylation.

## Abstract (web summary)

Current methods for production of monoclonal antibodies often require refolding steps or secretion across biological membranes. Here, Robinson *et al.* describe engineered *E. coli* strains for efficient production of functional immunoglobulin G antibodies in the bacterial cytoplasm.

## Graphical abstract

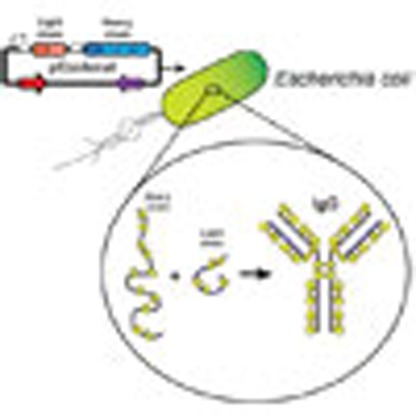

Over the past three decades, monoclonal antibodies (mAbs) have become one of the most useful protein tools with a myriad of diagnostic and therapeutic applications. For example, of the 151 unique recombinant therapeutics approved by the Food and Drug Administration, one-third of them are mAbs, with many more in research and development pipelines. At present, the majority of mAbs approved for therapeutic applications are produced in Chinese hamster ovary cells (12 out of 28), followed by SP2/0 (7/28) and NS0 (5/28) mouse cell lines, and hybridomas (2/28)^1^. The remaining two are antigen-binding fragments (Fabs) that are produced periplasmically in *Escherichia coli*.

With this expansion in the use of mAbs has come an increased demand for systems that enable rapid, cost-effective production and customization using molecular engineering. Mammalian cell expression systems offer a number of potential advantages for generating mAbs including high-level expression and stability^2^, and have recently been developed for the display of functional glycosylated immunoglobulin (Ig)Gs on the cell surface^3^,^4^. However, the selection of stable antibody-producing cell lines is very time consuming. In addition, despite the potential for high mAb yields in Chinese hamster ovary cells (∼10 g l^−1^ of culture), mammalian cell expression requires a long production cycle that results in higher cost of goods relative to microbial expression systems that involve much faster growth rates and thus lower capital investment^5^,^6^. Moreover, mammalian cell surface display has been hampered by the smaller library sizes that can be screened and the appearance of multiple copies of antibodies with different specificities on a single cell surface, making it difficult to directly identify and isolate antibodies with a desired property from naive libraries. In addition, with the increasing demand for animal-free products, alternative production platforms continue to be sought. Some possibilities include *in vitro* cell-free expression systems^7^, algae^8^, baculoviral insect^9^ or plant cells^10^ and *Drosophila melanogaster*^11^; however, each of these presents many of the same challenges faced with mammalian systems while also being much less utilized.

*E. coli* on the other hand remains a system of choice, finding broad use in both industry and academia for bench- and large-scale production of recombinant proteins. A major challenge facing the use of *E. coli* as an antibody expression platform is the production of mAbs with the correct disulfide bonds. The formation of disulfide bonds in *E. coli* can be catalysed in either the naturally oxidative periplasmic compartment^12^ or in the cytoplasm of genetically engineered strains^13^,^14^. Indeed, many fragments derived from mAbs such as Fab^15^, single-chain Fv (scFv)^16^, Fc^17^ and an scFv–Fv fusion^18^ have been expressed in the periplasm or in the cytoplasm of specially engineered *E. coli*^19^,^20^,^21^. There have even been two reports describing expression and functional assembly of full-length IgGs in *E. coli*, both in the periplasm^22^,^23^. However, periplasmic expression is thought to be limited by the smaller volume and the lack of adenosine triphosphate (ATP)-dependent molecular chaperones in this compartment, as well as by the need for extensive optimization to efficiently secrete both the IgG heavy chain (HC) and light chain (LC) across the tightly sealed cytoplasmic membrane. To address these limitations, several groups have attempted to produce soluble IgGs in the cytoplasm of *E. coli*; however, none have been successful^24^,^25^,^26^. At best, these efforts resulted in misfolded IgG chains within inclusion bodies, which required further *in vitro* refolding processes.

Here, we demonstrate that biologically active IgGs can be obtained by expression in the engineered oxidative cytoplasm of an *E. coli* strain called SHuffle^14^. In fact, significantly higher titres of cytoplasmic IgGs named ‘cyclonals' were obtained compared with periplasmic IgG expression, suggesting that membrane translocation is a limiting step in bacterial IgG production. Indeed, protein transport across biological membranes is energetically expensive and is often associated with negative pleiotropic effects^27^,^28^. And unlike the periplasm, which lacks ATP, the cytoplasm of *E. coli* harbours several energy-dependent folding chaperones (for example, GroEL, ClpXP and Hsp90) that may promote more efficient IgG folding and assembly. Further, we show that simple grafting of Fv domains from previously isolated IgGs permits on-demand production of entirely new cyclonals that bind specifically to diverse antigens. One concern, however, regarding the use of the cytoplasm is the absence of asparagine-linked (*N-*linked) glycosylation, which in the context of IgGs is necessary for *in vivo* effector function via binding to cognate Fcγ receptors (FcγRs)^29^,^30^ and for circulating half-life retention time^31^. We addressed this concern by modifying cyclonals with previously identified Fc mutations that endow IgGs with the ability to bind the receptors FcγRI, FcγRIIa, FcγRIIb and FcγRIIIa^32^,^33^,^34^. The end result is an entirely cytoplasmic system for efficient biosynthesis of immunologically and therapeutically relevant IgGs without the need for membrane translocation or glycosylation. This platform not only provides a powerful complement to the existing antibody expression toolkit, but should open the door to a range of applications such as the rapid conversion of phage-displayed scFvs into full-length IgGs or animal-derived IgGs into humanized clones.

## Results

### Cytoplasmic IgG production in SHuffle cells

**Figure 1:**
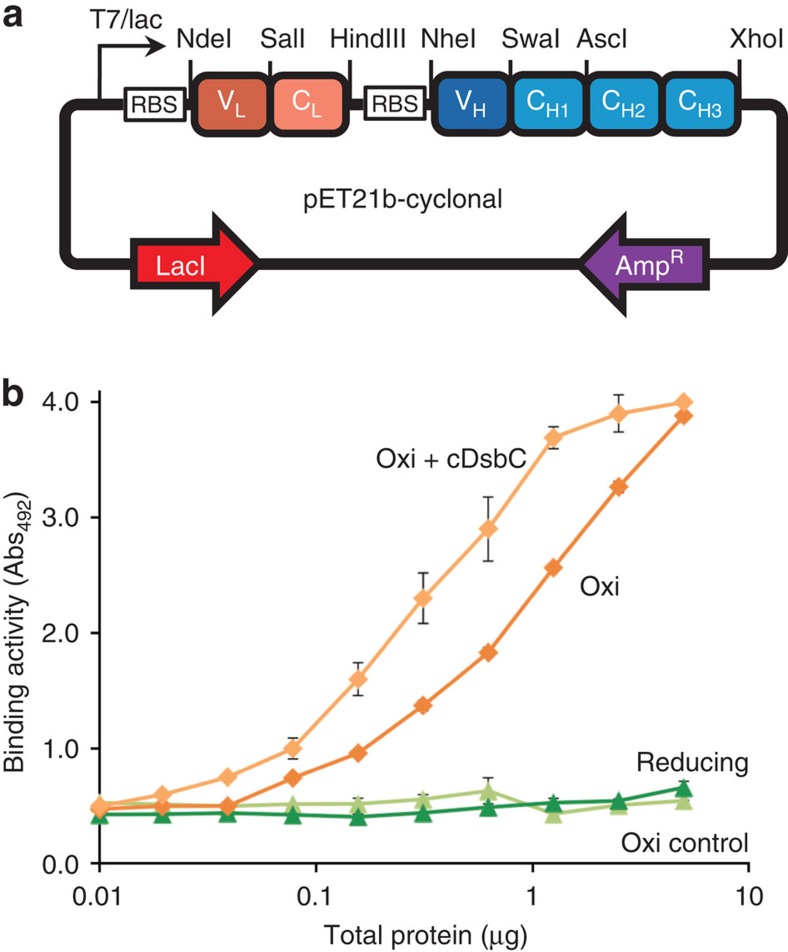
Cytoplasmic expression of mouse anti-MBP cyclonals in SHuffle. (**a**) Schematic of pET21b-based vector for expression of cyclonals in *E. coli*. V_H_, variable heavy; V_L_, variable light; C_H_, constant heavy; C_L_, constant light; RBS, ribosome-binding site. (**b**) ELISA signals (Abs_492_) for mouse anti-MBP cyclonals in lysates generated from the following: WT *E. coli* B strain with a reducing cytoplasm (reducing); isogenic *E. coli* strains MB1731 and SHuffle engineered with oxidizing cytoplasms (oxi); and, in the case of SHuffle, cytoplasmic DsbC (oxi+cDsbC). The activity measured in MB1731 cells carrying empty pET21b served as a negative control (oxi control). Data are expressed as the mean±s.e.m. of biological triplicates.

To enable production of cyclonals in *E. coli*, the genes encoding HC (V_H_–C_H_1–C_H_2–C_H_3; C_H_2–C_H_3 is a murine γ1 constant region) and LC (V_L_–C_L_; C_L_ is a murine *κ* constant region) were assembled into a synthetic, bicistronic operon under the control of a strong T7/lac promoter in plasmid pET21b (Fig. 1a). Our initial construct was generated using the V_H_ and V_L_ domains of an anti-maltose-binding protein (anti-MBP) antibody. The resulting plasmid was transformed in either a wild-type (WT) *E. coli* B strain or the isogenic *trxB gor* suppressor strain MB1731, whose cytoplasmic reductive pathways have been diminished, allowing the formation of disulfide bonds in the cytoplasm^13^,^14^ (Supplementary Fig. 1). Both strains carry a genomic copy of T7 gene1, which encodes the T7 RNA polymerase that permits expression of genes under the regulation of the T7 promoter. As expected, no IgG activity above background was observed in WT *E. coli* cells expressing the anti-MBP cyclonal (Fig. 1b), consistent with the earlier observations that IgGs do not fold correctly in a normal reducing cytoplasm^24^,^25^,^26^. In contrast, expression of the anti-MBP cyclonal in MB1731 cells resulted in a marked increase in IgG activity (Fig. 1b), indicating that an oxidizing cytoplasm is sufficient for the correct folding of full-length IgG.

IgG folding and assembly processes are dependent on multiple disulfide bonds^35^. Hence, we hypothesized that cyclonal production could be enhanced by expression of *E. coli* DsbC, an oxidoreductase chaperone capable of enhancing oxidative protein folding both in its native periplasmic compartment and when expressed cytoplasmically^13^,^19^,^36^. To test this notion, SHuffle T7 express cells (hereafter SHuffle), which are isogenic with MB1731 but carry a copy of *dsbC* that lacks its native signal sequence and is regulated by the *rrnB* promoter^14^, were transformed with the anti-MBP cyclonal-encoding plasmid. In the presence of cytoplasmic DsbC, cyclonal activity was measurably increased (Fig. 1b) without any significant difference in growth compared with MB1731 cells expressing the same cyclonal construct. In addition to mouse IgG, rabbit antibodies specific for two different human proteins, namely β2 microglobulin and prostate-specific membrane antigen, were actively expressed in the cytoplasm of SHuffle cells (Supplementary Fig. 2a). This is significant because rabbit mAbs are much more difficult to develop compared with mouse antibodies due to the lack of a stable fusion partner cell line for hybridoma development. Taken together, these results confirm that full-length IgGs can be produced as soluble proteins in the bacterial cytoplasm when the cytoplasmic reductive pathways have been diminished.

### Humanizing cyclonals via domain swapping

**Figure 2:**
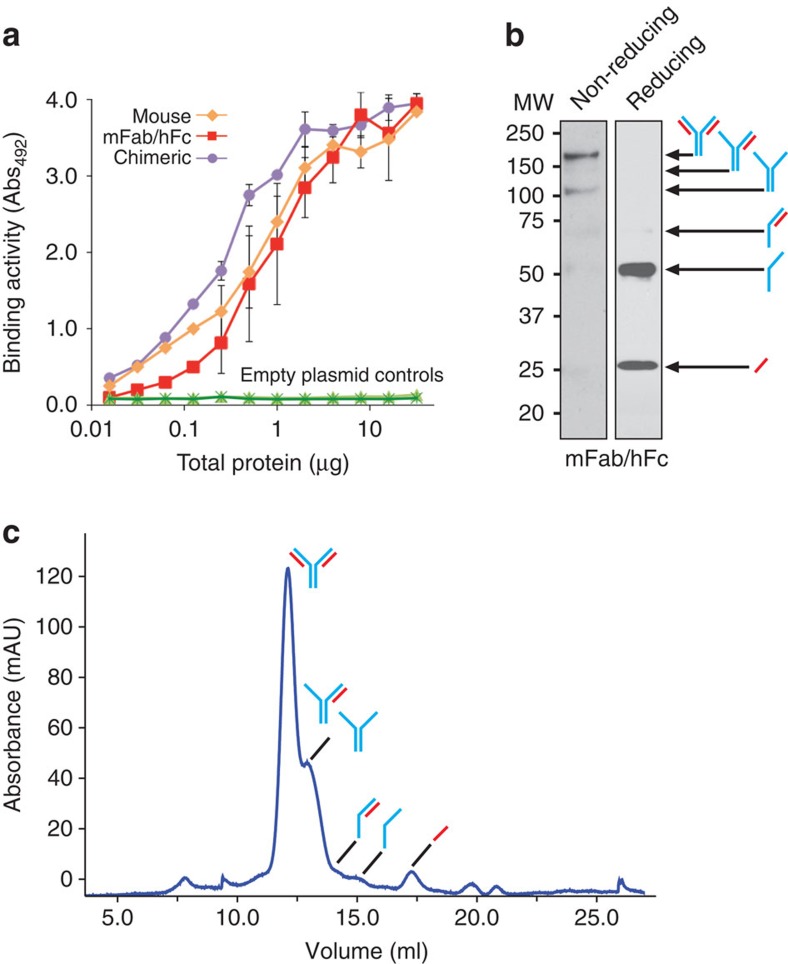
Expression of mouse-human hybrid cyclonals. (**a**) Antigen-binding activity for mouse, mFab–hFc hybrid, and chimeric anti-MBP cyclonals. ELISA signals (Abs_492_) for mouse cyclonal and empty plasmid control (light-green triangle) samples in cell lysates were obtained with anti-mouse antibodies; mFab/hFc cyclonal, chimeric cyclonal and corresponding empty plasmid control (dark-green star) were detected in cell lysates with anti-human Fc antibodies. Data is expressed as the mean±s.e.m. of biological triplicates. (**b**) Soluble anti-MBP cyclonal in the mFab–hFc format was purified from cell lysates prepared from SHuffle by protein-A affinity chromatography and analysed by western blot under non-reducing (left panel) and reducing (right panel) conditions. Arrows indicate fully assembled cyclonal as well as other intermediate species. The percentage of fully assembled heterotetrameric product among all products was 63±5% (left panel) as determined by densitometry analysis. (**c**) Representative SEC analysis of protein-A-purified anti-MBP cyclonal in the mFab/hFc format performed using a Superdex 200 10/300 GL gel filtration column. Heterotetramer and other intermediate products are labelled.

Human, or human hybrid, antibodies are typically less immunogenic than those of mice^37^; therefore, it has become a common practice to ‘humanize' mouse antibodies. Humanizing involves combining the antigen-binding portions of a mouse antibody with the constant regions of a human antibody using recombinant DNA techniques. To create humanized cyclonals, we re-engineered the mouse anti-MBP cyclonal in two ways. First, the mouse anti-MBP Fabs were spliced onto human Fc resulting in a mouse Fab/human Fc (mFab/hFc) hybrid. Second, the mouse anti-MBP variable domains were fused to human constant domains (mouse V_L_ to human C_L_ and mouse V_H_ to human C_H_1–C_H_2–C_H_3 for LC and HC, respectively) resulting in a chimeric antibody that contained only ∼30% mouse sequence as described previously^38^,^39^. Both newly engineered cyclonals were expressed as functional IgGs in the cytoplasm of SHuffle cells at a level that was comparable to that of the mouse cyclonal (Fig. 2a). Western blot analysis under non-reducing conditions revealed that nearly 70% of the HC was associated with the LC in fully assembled, heterotetrameric IgGs (Fig. 2b, shown for the mFab/hFc hybrid). Size-exclusion chromatography further confirmed the high efficiency of heterotrimer formation for the SHuffle-derived anti-MBP cyclonal (Fig. 2c). Similar domain swapping with rabbit cyclonals resulted in rabbit Fab/human Fc (rFab/hFc) hybrids that bound cognate antigens as effectively as their progenitors (Supplementary Fig. 2a). In addition, a cyclonal version of trastuzumab (Herceptin), an Food and Drug Administration-approved humanized IgG that selectively targets the extracellular domain of the human epidermal growth factor receptor 2 (HER2/neu) and is only ∼10% mouse in sequence^40^, was also observed to efficiently bind the antigen (Supplementary Fig. 2b). Hence, functional IgGs comprised predominantly of human sequences were readily obtained using the cyclonal expression platform.

Next, the bacterially produced cyclonals were compared with mammalian cell-derived IgG produced using conventional hybridoma cell culture. With respect to antigen binding, purified cyclonals in the mFab/hFc and mouse formats bound soluble MBP in enzyme-linked immunosorbent assay (ELISA) with similar avidity to the corresponding hybridoma-derived IgG (Supplementary Fig. 3a). Cyclonals also performed comparably to the hybridoma IgG when each was used in a western blot format (Supplementary Fig. 3b). With respect to stability, we compared the serum stability at 37 °C of cyclonals in the mFab/hFc and mouse formats with that of hybridoma-derived IgGs. Importantly, all IgGs were equally stable, losing little to no binding activity over the test period of 4 days (Supplementary Fig. 3c). Hence, the activity of antibodies produced in the cytoplasm of bacteria is on par with those produced recombinantly in mammalian cells.

### Variable region grafting yields new cyclonal specificities

**Figure 3:**
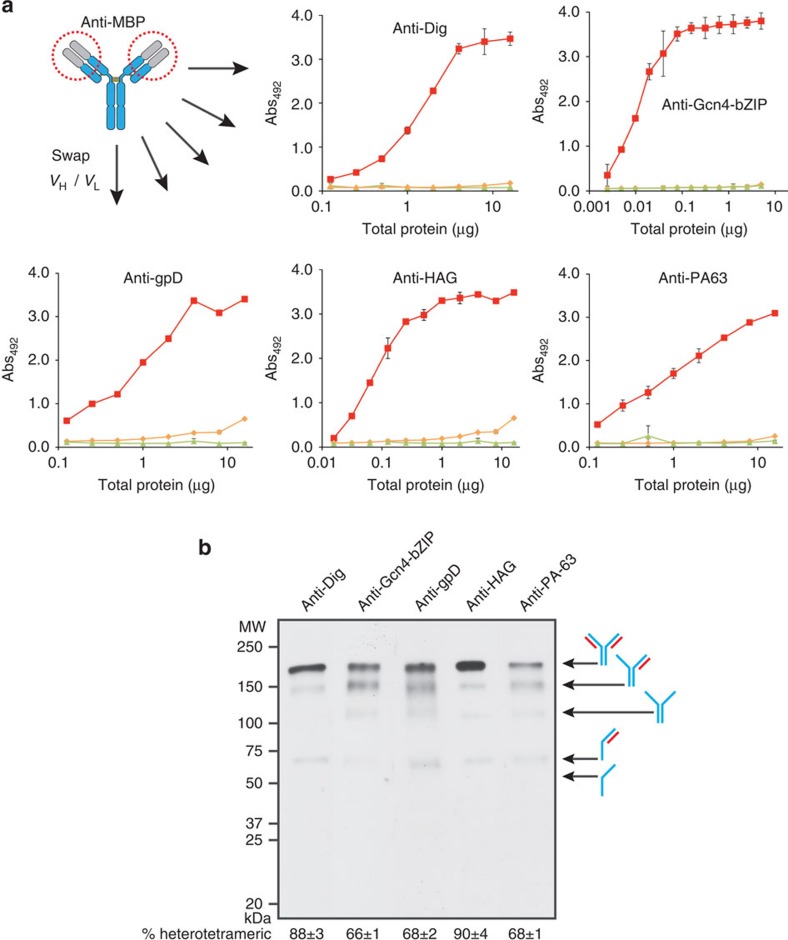
Redirecting cyclonals to new antigens with swapped variable regions. (**a**) Antigen-binding activity in SHuffle lysates for anti-MBP cyclonals with swapped V_H_ and V_L_ domains with specificity for antigens as indicated. ELISA signals (Abs_492_) for mFab/hFc cyclonals (red), parental anti-MBP cyclonal (orange) and empty plasmid control (green) in cell lysates were obtained with anti-human Fc antibodies. Data are expressed as the mean±s.e.m. of biological triplicates. (**b**) Representative non-reducing western blot of different cyclonals in the mouse Fab-human Fc format following protein-A affinity purification from cell lysates prepared from SHuffle cells. Arrows indicate fully assembled cyclonal and other HC intermediate species. The percentage of fully assembled product (% heterotetrameric) among all products was determined for each cyclonal using densitometry analysis. Percentages are expressed as the mean±s.e.m. of biological triplicates.

Because of the ease with which new genes can be designed, cloned and expressed in *E. coli*, the cyclonal platform affords the unique opportunity for on-demand production of customized antibodies. To demonstrate this concept, we created a panel of new cyclonals in a process that required less than a week to complete. This involved first replacing the V_H_ and V_L_ genes of the anti-MBP cyclonal with the same genes from a variety of existing antibodies and antibody fragments including: YMF10, a chimeric IgG specific for proteolytically processed and heptamerized *Bacillus anthracis* protective antigen (PA-63)^22^; 26.10-IgG, a murine IgG specific for digoxin (Dig)^22^; D10, an scFv specific for the capsid protein D (gpD) of bacteriophage lambda^41^; anti-Gcn4 scFv (Ω-graft variant) that binds the 47-residue bZIP domain of the yeast transcription factor Gcn4 (Gcn4-bZIP)^42^; and h6-4, an scFv that binds a six-residue peptide derived from haemagglutinin (HAG) of influenza virus^43^. Following expression in SHuffle cells, cyclonals in the mFab/hFc format were observed to bind their cognate antigens with high affinity (Fig. 3a). Importantly, the parental anti-MBP cyclonal showed no significant binding activity against the new antigens (Fig. 3a) and none of the grafted cyclonals recognized MBP (Supplementary Fig. 4), confirming a complete change in specificity by simple swapping of the variable domains. Western blot analysis under non-reducing conditions revealed fully assembled, heterotetrameric IgGs in each case with the percentage of fully assembled heterotetrameric product among all products ranging from 66 to 90% under the conditions tested (Fig. 3b). We routinely purified ∼1–25 mg of highly active IgGs per litre of shake-flask culture using affinity chromatography on a protein-A column (Supplementary Fig. 5a,b). It is also worth mentioning that one of the grafted cyclonals (anti-Gcn4-bZIP) and its progenitor scFv clone both exhibited nanomolar equilibrium dissociation constants as determined by Biacore analysis (Supplementary Fig. 6; *K*_d_=5.5 nM for cyclonal versus 0.5 nM for scFv), confirming the feasibility of scFv-to-IgG reformatting in the cyclonal context.

### Remodelling the Fc domain of cyclonals for binding to FcγRs

**Figure 4:**
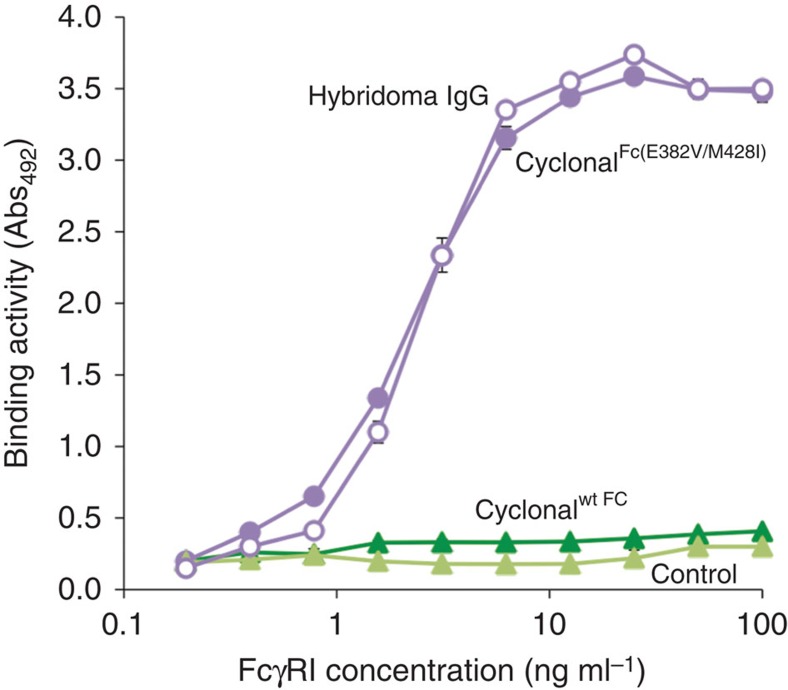
Binding of FcγRI by cyclonals with remodelled Fc domains. Antigen-binding activity of chimeric anti-PA-63 cyclonal with either WT or mutated Fc domain (cyclonal^Fc(E382V/M428I)^). Glycosylated IgGs with WT Fc were purified from hybridoma cultures and included as positive control. Equivalent amounts of purified *E. coli* cyclonals or hybridoma IgGs were used to coat ELISA plates and purified FcγRI was applied at various concentrations as indicated. Blank buffer was used as a control. Data is expressed as the mean±s.e.m. of biological triplicates.

IgGs lacking glycosylation in their Fc domain, such as those produced in *E. coli*, are completely unable to bind to FcγRs, and therefore do not induce FcγR-mediated effector functions^23^,^44^. However, aglycosylated Fc variants that productively engage the receptors FcγRI (CD64), FcγRIIa (CD32a), FcγRIIb (CD32b) and FcγRIIIa (CD16a) with moderate-to-high affinity have been isolated^32^,^33^,^34^. In the case of FcγRI, binding was critically dependent on the amino-acid substitution E382V, and to a lesser extent M428I, within the C_H_3 domain. To engineer cyclonals that bind FcγRs, we introduced the E382V and M428I mutations to the human Fc domain of the chimeric anti-PA-63 cyclonal. The WT and E382V/M428I variant cyclonals were each expressed in the cytoplasm of SHuffle cells and purified by protein-A affinity chromatography. As expected, only the E382V/M428I variant exhibited binding of FcγRI that was significantly above background and on par with the binding obtained with glycosylated IgGs produced by hybridoma cells (Fig. 4). These results confirm that the activity conferred by Fc remodelling is maintained following expression in the bacterial cytoplasm and that *in vitro* FcγRI binding by cyclonals rivals that of antibodies produced by mammalian cell culture.

### Comparison of cytoplasmic versus periplasmic IgG expression

**Figure 5:**
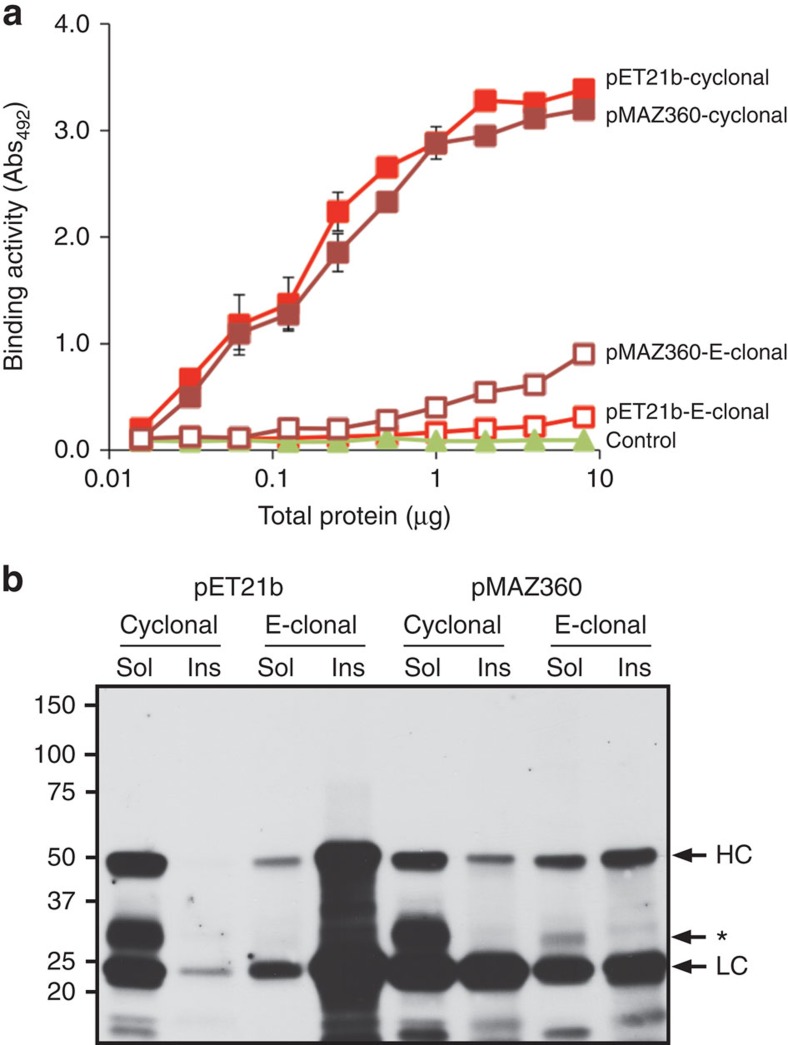
Comparison of cytoplasmic versus periplasmic IgG expression. (**a**) Antigen-binding activity of mFab/hFc anti-MBP cyclonals derived from the cytoplasm of MB1731 cells (closed squares) or E-clonals derived from the periplasm of parental B strain (open squares). Expression was either from plasmid pET21b (red) or pMAZ360 (dark red); signal from cells carrying empty plasmid was used as negative control (green). Data are expressed as the mean±s.e.m. of biological triplicates. (**b**) Western blot analysis of soluble (sol) and insoluble (ins) fractions derived from the same cells as in **a**. HC, heavy chain; LC, light chain; asterisk, degraded HC. Mass spectrometry analysis of the degraded HC revealed that the cleavage site is in the CH1 domain of HC, known to fold inefficiently and be the major limiting factor in the assembly of IgG in eukaryotic cells. This bottleneck seems to carry over to SHuffle cells and is likely due to site-specific cleavage of misassembled HC by cytoplasmic protease(s).

It has been reported that secretory production of functional IgGs in the periplasm is inefficient and could only be achieved by lowering protein translation rates^23^. This observation combined with the large energetic cost associated with moving polypeptides across biological membranes^27^ led us to hypothesize that the exceptional expression levels achieved in the cytoplasm of SHuffle cells might be due to the elimination of the membrane translocation step and/or access to ATP-dependent chaperone systems. To test this hypothesis, we directly compared the accumulation of the anti-MBP IgG in the mFab/hFc format following expression in the cytoplasm and periplasm. For periplasmic expression, we generated a bicistronic construct in pET21b where the HC and LC of the anti-MBP IgG were both fused to the *pelB* signal peptide, yielding a so-called ‘E-clonal'^22^ specific for MBP. Unlike the periplasm of WT cells, SHuffle cells overexpress a cytoplasmic version of DsbC that could unfairly bias the cytoplasmic versus periplasmic analysis. Therefore, for this comparison we used the SHuffle progenitor strain MB1731 to express all cyclonal constructs, because it has an oxidizing cytoplasm but lacks a cytoplasmic copy of *dsbC.* When overexpressed from a T7 promoter, the antigen-binding activity for the anti-MBP E-clonal was barely above that measured for cells carrying an empty plasmid, regardless of whether we used MB1731 cells or the WT B strain that was isogenic with MB1731 but with a reducing cytoplasm^14^ (Fig. 5; shown for WT B strain). In stark contrast, expression of the anti-MBP cyclonal in isogenic MB1731 cells resulted in a marked increase in antigen-binding activity, reaching a level that was ∼10-fold greater than its E-clonal counterpart (Fig. 5a). Western blot analysis indicated that the HC and LC of the anti-MBP cyclonal accumulated almost exclusively in the soluble fraction, whereas periplasmic targeting of the E-clonal HC and LC resulted in significant accumulation in the insoluble fraction with only slight accumulation in the soluble fraction (Fig. 5b). Since expression from T7-based plasmids can often lead to this type of severe insoluble accumulation, we decided to test an alternative expression plasmid, pMAZ360, that was previously shown to be optimized for E-clonal production^22^. When the anti-MBP E-clonal construct was expressed from pMAZ360, where expression was driven from a *lac* promoter, a modest increase in activity was observed (Fig. 5a). In line with this increased activity, we observed greater accumulation of the E-clonal HC and LC in the soluble fraction; however, a significant amount still partitioned to the insoluble fraction (Fig. 5b). As above, expression of the anti-MBP cyclonal from pMAZ360 was significantly greater than expression of the E-clonal from the same plasmid (Fig. 5a). In fact, cyclonal expression from pMAZ360 was indistinguishable from pET-based expression. Despite this high activity, the cyclonal HC and LC accumulated in the insoluble fraction following pMAZ360 expression at a level that was similar to the E-clonal chains (Fig. 5b). Nonetheless, soluble accumulation of the cyclonal HC and LC was significantly higher compared with that of the E-clonal, especially in the case of the HC. Nearly identical results were obtained when comparing cytoplasmic and periplasmic expression of the anti-HAG and anti-PA-63 IgGs from pET21b and pMAZ360 (Supplementary Fig. 7) and Herceptin from pMAZ360 (Supplementary Fig. 3b), indicating that the advantage of cytoplasmic expression was not confined to the anti-MBP IgG construct.

## Discussion

We report the first soluble expression of active, full-length IgGs in the cytoplasm of SHuffle *E. coli* cells, a feat that was previously unattainable using WT *E. coli* cells whose cytoplasmic compartments were not engineered for disulfide bond formation^24^,^25^,^26^. This advance taps the wealth of knowledge and comprehensive tools (for example, tightly regulated promoters, chaperone co-expression systems) available for this host, making it possible to produce large quantities of recombinant antibodies for *in vitro* diagnostic techniques as well as *in vivo* therapeutic applications. Moreover, we show that expression in the SHuffle cytoplasm can be combined with standard molecular engineering strategies such as grafting alternative epitope recognition domains, humanizing^37^,^38^,^39^ and Fc engineering^23^,^44^ to rapidly (∼1 week or less) create custom mouse, rabbit or humanized antibodies exhibiting clinically relevant properties. These results are in direct agreement with the recent observation that engineered biophysical properties can be readily transferred between different antibody formats and expression systems^45^. Hence, we anticipate that the cyclonal expression technology described here will find use in several applications such as rapidly converting phage-displayed scFvs into full-size IgGs or molecular reformatting mouse- or rabbit-derived IgGs into humanized analogues.

A significant limitation in the therapeutic use of IgGs expressed from prokaryotes is the lack of post-translational glycosylation of the Fc domain. Specifically, *N*-linked glycosylation at the conserved N297 residue is necessary for efficient binding of the Fc domain with its cognate FcγRs. Upon binding, effector function is activated resulting in antibody-dependent cell-mediated cytotoxicity or antibody-dependent cell-mediated phagocytosis or complement-dependent cytotoxicity^30^. We have bypassed this limitation by remodelling cyclonal Fc domains with previously discovered mutations (for example, E382V/M428I) that permit efficient binding of aglycosylated IgG to FcγRs^33^. The result is a synthetic pathway, entirely in the *E. coli* cytoplasm, for producing IgGs that bind specific antigens and also engage FcγRs with the potential to activate immune cells. In fact, bacterially produced cyclonals equalled the performance of the same IgGs produced using conventional mammalian cell culture (that is, hybridomas) in both antigen- and FcγR-binding properties.

A major advantage of cytoplasmic expression is the elimination of the membrane translocation step, which is a prerequisite for soluble antibody production in all other cell-based systems. By circumventing the physical membrane barrier, we observed much greater soluble accumulation and activity for two different cyclonals, independent of the plasmid backbone choice, compared with their periplasmic counterparts. In particular, the expression of cyclonals but not periplasmic IgGs was harmonious with pET-based plasmids, which are well known for their high levels of protein expression. It should be noted that this high level of expression was achieved following direct cloning into the multi-cloning site of standard plasmids and did not require any additional gene-level optimization, even in the face of major changes to variable and framework regions. In contrast, to achieve soluble IgG expression in the *E. coli* periplasm required screening of a panel of translation initiation regions (TIRs) to balance expression of each polypeptide chain and lower the protein translation rate^23^. The lower translation rate is thought to decrease protein load on the Sec pathway and also ensure that protein substrates remain unfolded for efficient secretion. Indeed, although not reported for IgGs specifically, when large amounts of complex hybrid proteins (for example, the signal sequence of outer membrane protein LamB fused to LacZ) are made, rapid folding of the hybrid in the cytoplasm can cause a lethal jamming of the Sec translocase^46^. Importantly, all of these secretion-related problems are avoided with cytoplasmic expression.

Another major advantage to expressing proteins in the cytoplasm is the presence of the many energy-dependent chaperones that facilitate correct folding of proteins, which are absent in the periplasm (for example, GroEL/S, ClpXP and DnaKJ systems). Such chaperone systems may be responsible for the significant cyclonal titres (∼1–25 mg l^−1^) observed here. By way of comparison, yields of periplasmically expressed IgGs produced in shake flasks rarely exceed 1 mg of assembled full-length antibodies per litre of culture with literature values of 0.004–0.008 (ref. 47), 0.1–1.3 (ref. 22) and 1–4 mg l^−1^ (ref. 48). In fact, cyclonal titres were typically greater than other less complex immunoglobulin formats, regardless of the whether they were expressed in the periplasm (for example, 0.2 mg l^−1^ Fv^49^; 2 mg l^−1^ Fab^50^) or the cytoplasm of redox-engineered *E. coli* (for example, 1–2 mg l^−1^ scFv^19^; 3–4 mg l^−1^ Fab^20^). This may be due to the cytoplasmic copy of DsbC in SHuffle cells, which was absent in earlier cytoplasmic expression systems. Indeed, levels of active cyclonal increased by ∼70% when expressed in the presence of cytoplasmic DsbC compared with an isogenic strain that lacked cytoplasmic DsbC (see Fig. 1). Taken together, our findings confirm the capacity of the cytoplasm as a compartment for high-titre IgG production.

Looking forward, many remaining factors known to be important for IgG expression and folding in other systems remain to be optimized for cyclonals. For example, we co-expressed a set of helper proteins that were previously shown to improve the production of disulfide-bonded proteins in SHuffle cells^14^. Several of these helpers including the yeast protein disulfide isomerase (PDI) homologue (MPD1) and a thioredoxin variant with a mutation in its characteristic CPHC active site motif enhanced the levels of active cyclonal (Supplementary Table 1). In light of these preliminary results, we suspect that co-expression of other endoplasmic reticulum-folding factors could further augment the accumulation of full-length cyclonals. Another strategy for improving cyclonal production titres is to toggle the ratio of LC and HC in the cytoplasm in a manner that favours the formation of fully assembled IgGs. Such toggling could be achieved by either modifying the sequences that control HC and LC expression (for example, TIRs and promoters) or altering the primary sequence of the IgG directly through the use of alternative frameworks and/or constant region sequences (that is, IgG isotypes). In one notable example, Yansura *et al.* showed that HC and LC levels in the *E. coli* periplasm could be more efficiently assembled using different-strength TIRs for L and HCs^23^. It is therefore conceivable that even higher titres of cyclonals can be reached in the future following further host and process optimization.

## Methods

### Bacterial strains and growth conditions

The bacterial strains used in this study are listed in Supplementary Table 2. A single colony of SHuffle T7 Express transformed with one of the pMAZ360–cIgG expression vectors was used to inoculate 5 ml Luria–Bertani (10 g l^−1^ tryptone, 5 g l^−1^ yeast extract, 5 g l^−1^ NaCl and NaOH to pH 7.2) supplemented with 100 μg l^−1^ carbenicillin and 25 μg l^−1^ spectinomycin, and grown overnight at 30 °C. The next day, 300 ml of fresh LB supplemented with 100 μg ml^−1^ carbenicillin was inoculated 1/100 with the overnight culture and cells were grown at 30 °C until reaching an absorbance at 600 nm (Abs_600_) of 0.7. At this point, cyclonal expression from the pMAZ360–cIgG vector was induced by addition of 1.0 mM isopropyl β-D-thiogalactopyranoside, after which cells were incubated an additional 16 h at 30 °C. Cells were harvested by centrifugation before preparation of lysates or purification of IgGs.

### Immunization, cell fusion and selection of hybridoma

A protein fusion consisting of bacteriophage M13 pIII (residues 259–406) and MBP was constructed in the pMAL expression system, expressed and purified according to the manufacturer's protocols (New England Biolabs). Two female BALB/c mice (Jackson Laboratories) were immunized intraperitoneally over a period of 4 months with a primary injection containing 50 μg antigen emulsified in Freund's complete adjuvant, followed by three subsequent boosts in Freund's incomplete adjuvant. A final tail vein injection containing 25 μg of antigen in 50 μl of sterile saline was administered 3 days before to removing the spleen and fusion of splenocytes with X63Ag8.653 parental myeloma cells^51^. Splenocytes were fused with X63Ag8.653 myelomas using the ClonaCell-HY Hybridoma Cloning Kit (StemCell Technologies Inc.). Hybridoma tissue culture supernatants (TCS) were screened initially by ELISA with MBP as antigen. The isotype of mAbs was determined using an antigen-capture ELISA. Wells were coated with anti-mouse immunoglobulin antibody (American Qualex International). Undiluted hybridoma tissue culture supernatant was used for isotyping. The isotype of each captured mAb was then determined using goat anti-isotype-specific alkaline phosphatase-conjugated antibodies (Caltag).

### Plasmid construction

A list of plasmids used in this study is given in Supplementary Table 2. To generate the initial anti-MBP IgG construct, total RNA was isolated from 4 × 10^7^ hybridoma cells using a Qiagen RNeasy kit with the following modifications: 4 × 10^7^ cells were resuspended in 600 μl RLT buffer, homogenized with hand-held homogenizer for 2 min followed by the addition of 1,800 μl RLT buffer (2,400 total, 600 μl per 1 × 10^7^ cells), mixed, separated into 4 × 600-μl aliquots and homogenized for another 30 s. Total RNA for each sample was purified separately according to protocol, eluted 2 × 30 μl for each sample and pooled (240 μl total). A mixture of degenerate forward primers (Deg1–Deg7, Supplementary Table 3) was designed from κ-chain sequences in the Kabat database^52^ and used with the 3′ reverse primer LC-9 (Supplementary Table 3) to amplify the complementary DNA (cDNA) sequence of the IgG κ-LC gene. Fifty -microlitre PCR mixtures for κ-LC cDNA amplification with Deep Vent DNA polymerase (New England Biolabs) contained buffer supplied by the manufacturer supplemented with 200 μM deoxynucleotide (dNTP) solution mix, 0.8 μM forward primer mixture, 4 μM reverse primer and ∼300 ng cDNA. Thermocycling consisted of incubation at 94 °C for 5 min followed by 30 cycles of successive incubations at 94 °C for 30 s, 55 °C for 30 s and 72 °C for 60 s. After thermocycling, a final extension was performed at 72 °C for 10 min. The amplified product was digested with EcoRI and cloned between the EcoRI–SmaI sites of pNEB193, resulting in plasmid pNEB–LC–MBP. To amplify the cDNA sequence of the IγG HC, a degenerate primer pair (Kabat-F and Kabat-R, Supplementary Table 3) were designed from IgG_2A_-chain sequences from the Kabat database^52^. PCR mixtures for IγG HC cDNA amplification with Taq DNA Polymerase (New England Biolabs) contained buffer supplied by the manufacturer supplemented with 200 μM dNTP solution mix, 4 μM each of forward and reverse primers and ∼300 ng cDNA. Thermocycling consisted of incubation at 94 °C for 5 min, 80 °C for 60 s, followed by 30 cycles of successive incubations at 94 °C for 30 s, 52 °C for 30 s and 72 °C for 2 min. After thermocycling, a final extension was performed at 72 °C for 10 min. The amplified product was digested with EcoRI and NotI and cloned between the EcoRI and NotI sites of pNEB193, resulting in plasmid pNEB–HC–MBP. Sequencing of this plasmid was used to design a synthetic HC gene in pUC57 (GenScript; pUC57-HC-MBPsyn) that was modified with unique internal restriction sites for future cloning.

The construction of all bacterial IgG expression plasmids and the reagents used in their construction (for example, DNA templates, primers and restriction enzymes) are described in Supplementary Tables 3 and 4. Briefly, the anti-MBP HC and LC lacking native export signals were PCR-amplified from pNEB-LC-MBP and pUC57-HC-MBPsyn, respectively, and subsequently assembled as a bicistronic operon using overlap extension PCR. This operon was initially cloned in plasmid pMAZ360–IgG^22^ and then subcloned into pET21b. The resulting plasmid, pET21b–cyclonal–MBP, placed expression of the HC and LC under the control of the strong T7/lac promoter (Fig. 1a). Construction of pET21b–cyclonal–MBP(mFab/hFc) involved double digestion of pET21b–cyclonal–MBP with AscI/XhoI to remove the mouse C_H_2 and C_H_3 domains, followed by ligation of DNA encoding human C_H_2 and C_H_3 domains between the same sites. Construction of pET21b–cyclonal–MBP(chimeric) involved double digestion of pET21b–cyclonal–MBP(mFab/hFc) with SalI/HindIII to remove the mouse C_L_ followed by ligation of DNA encoding human C_L_ between the same sites. To generate mFab/hFc cyclonals with new antigen specificity, V_L_ domain sequences from existing antibodies and antibody fragments^22^,^41^,^42^,^43^ were amplified by PCR, while in parallel, mouse C_L_ region DNA was also amplified by PCR. During PCR, engineered sequence overlaps were introduced into both the V_L_ and C_L_ sequences. The two PCR products were combined by overlap extension PCR and the resulting product was cloned between NdeI and HindIII of pET21b–cyclonal(mFab/hFc). Corresponding V_H_ domain sequences from the same existing antibodies and antibody fragments were amplified by PCR, while in parallel, mouse C_H_1 region DNA was also amplified by PCR. During PCR, engineered sequence overlaps were introduced into both the V_H_ and C_H_1 sequences. The two PCR products were combined by overlap extension PCR, and the resulting product was cloned between NheI and AscI of pET21b-cyclonal(mFab/hFc). For chimeric constructs with new antigen specificity, PCR-amplified V_L_ domain sequences from existing antibodies and antibody fragments were cloned directly between NdeI and SalI of pET21b–cyclonal(chimeric). Corresponding V_H_ domain sequences were assembled with human C_H_1 region DNA by overlap extension PCR and the resulting products were cloned between NheI and AscI of pET21b-cyclonal(chimeric). The E382V Fc mutation^28^ was introduced into the C_H_3 sequence of the chimeric anti-PA-63 HC by overlap extension PCR followed by a nested PCR using a mutagenic reverse primer to generate the M428I Fc substitution^28^. The resulting mutant HC was ligated between NheI and XhoI of pET21b-cyclonal–PA-63(chimeric). To construct E-clonal expression plasmids, DNA encoding the *E. carotovora* pectate lyase signal peptide (spPelB) was fused to the 5′ end of the HC and LC genes from anti-MBP (mFab/hFc) using nested PCR. An ribosome-binding site was introduced upstream of the spPelB–HC fusion in the same manner. The resulting spPelB–HC and spPelB–LC sequences were cloned between the NdeI and HindIII and HindIII/XhoI sites, respectively, of pET21b–cyclonal–MBP, yielding pET21b-E-clonal–MBP(mFab/hFc). All subsequent periplasmic constructs were synthesized by inserting the appropriate LC and HC pairs into pET21b-E-clonal-MBP(mFab/hFc) using the NcoI/HindIII and NheI/XhoI sites, respectively. To generate additional pMAZ360 expression constructs, a synthetic multi-cloning site was cloned between NdeI and AscI of pMAZ360 (ref. 22) such that the same restriction sites used in synthesizing the bicistronic pET21-cyclonal and pET21-E-clonal plasmids could also be used to generate pMAZ360-cyclonal and pMAZ360-E-clonal expression vectors coding for any of the different IgGs. For construction of rabbit antibodies, HC and LC genes from rabbit anti-prostate-specific membrane antigen and anti-β2 microglobulin clones (kindly provided by Cell Signaling Technology) were PCR amplified and cloned between NcoI and BamHI of pETDuet-1 (Novagen). Construction of rFab/hFc hybrids in pETDuet-1 was performed using overlap extension PCR as described above for mFab/hFc cyclonals. Construction of Herceptin in the cyclonal format was performed by first PCR amplifying the gene encoding the LC from pSTJ4-AglycoT^33^ and cloning the PCR product between the NdeI and HindIII sites of pMAZ360–cIgG–aMBP. Next, DNA encoding the HC was amplified from pSTJ4-AglycoT and cloned between the NheI and XhoI sites of pMAZ360–cIgG, resulting in the vector pMAZ360–cIgG–Herceptin. Genes encoding the target antigens bacteriophage lambda gpD^41^, the 47-residue bZIP domain of the yeast transcription factor Gcn4 (ref. 42) and the 6-residue peptide derived from HAG of influenza virus^43^ were each cloned into pET28a(+), which introduced a 6 × -His tag to the N terminus of each antigen. Due to their relatively small sizes, Gcn4-bZIP and HAG were fused to the C terminus of glutathione *S-*transferase (GST). All plasmids constructed in this study were confirmed by sequencing.

### Preparation of soluble and insoluble fractions

SHuffle cells expressing cyclonals were harvested by centrifugation at 13,000*g* and 4 °C for 30 min. Harvested cells were resuspended in a volume of PBS, 1 × SigmaFASTprotease inhibitor cocktail (EDTA free) and 5 mM EDTA equal to one-tenth of the original culture volume. Cells were ruptured by passage through an Emulsiflex-C5 High Pressure Homogenizer at 16,000–18,000 psi. The cell lysate was then clarified by centrifugation at 30,000*g* and 4 °C. The supernatant containing soluble cytoplasmic proteins was recovered as the soluble fraction and then passed through a 0.2-μm sterile membrane filter. Insoluble pellets were washed three times in 50 mM Tris-HCl, 1 mM EDTA (pH 8.0) and then resuspended in PBS supplemented with 2% SDS. Inclusion bodies were solubilized by heating at 100 °C for 10 min. The solubilized mixture was centrifuged at 16,000*g* for 10 min and the supernatant was collected as the insoluble fraction.

### Protein purification

For cyclonal purification, protein-A agarose resin was equilibrated with 10 ml PBS and then mixed with the filtered soluble lysate fraction. The resin-soluble lysate fraction mixture was incubated at room temperature with end-over-end mixing for 2 h. The mixture was then applied to a polypropylene gravity column and the soluble lysate was allowed to completely pass through the column. After settling in the column, the protein-A agarose was washed with 25 ml PBS. Cyclonals were eluted from the column with 0.1 M glycine-HCl (pH 3.0) in 1-ml fractions and neutralized with 100 μl 1 M Tris (pH 9.0). Purified fractions were resolved by SDS–polyacrylamide gel electrophoresis under non-reducing conditions and either visualized by staining with Coomassie Blue G-250 or further processed for western blot as described below.

### Western blot analysis

For SDS–polyacrylamide gel electrophoresis analysis under non-reducing conditions, samples were diluted 1:1 in 2 × Laemmli sample buffer. For reducing conditions, samples were diluted 1:1 in 2 × Laemmli sample buffer supplemented with 2.5% 2-mercaptethanol. In both cases, samples were heated at 100 °C for 10 min and then loaded on 4–20% tris-glycine gels (Bio-Rad). All samples were normalized by total protein, as determined by the Bradford assay. Following electrophoresis, resolved proteins were transferred to polyvinylidene fluoride membranes (Millipore). Membranes were rinsed with PBS and then blocked with 5% milk (w/v) in PBS containing 0.05% Tween-20 (PBST) for 1 h. After three washes with PBST, membranes containing mFab/hFC or chimeric IgGs were probed with 1:7,500-diluted anti-human Fc-horseradish peroxidase (HRP) conjugate antibodies (Thermo Scientific) plus 1:10,000-diluted anti-mouse Igκ chain-HRP conjugate antibodies (Jackson ImmunoResearch) in 2% milk (w/v)-PBST for 1 h. After washing six more times with PBST, membranes were incubated with Immun-star HRP substrate (Bio-Rad) and then visualized using a Bio-Rad Chemidoc XRS+ or by exposing the membranes to X-ray film.

### ELISA

Costar 96-well ELISA plates (Corning) were coated overnight at 4 °C with 50 μl of 4–10 μg ml^−1^ antigen in 0.05 M sodium carbonate buffer (pH 9.6). Antigens used were MBP5 (New England Biolabs), digoxin-bovine serum albumin (Fitzgerald Industries), PA-63 (Calbiochem), as well as gpD, GST-Gcn4 and GST-HAG that were each expressed in *E. coli* T7 express cells and purified by standard nickel-charged affinity resin (Ni-NTA) purification. After blocking with 3% (w/v) milk in PBST for 1–3 h at room temperature, the plates were washed four times with PBS buffer and incubated with serially diluted purified IgG samples or soluble fractions of crude cell lysates for 1 h at room temperature. IgG-containing samples were quantified by the Bradford assay and an equivalent amount of total protein (typically 8–64 μg) was applied to the plate. After washing four times with the same buffer, 50 μl of the following antibodies in 3% PBST was added to each well for 1 h: 1:4,000-diluted goat anti-mouse IgG (H+L)-HRP conjugate (Promega) or anti-mouse IgG (Fab specific)-HRP conjugate (Sigma) for mouse IgGs; 1:5,000-diluted rabbit anti-human IgG (Fc) antibody–HRP conjugate (Pierce) for chimeric and mFab/hFc IgGs; 1:5,000-diluted goat anti-rabbit IgG (H+L)-HRP conjugate (Pierce) for rabbit IgGs; and 1:5,000 anti-human IgG (H+L)-HRP conjugate (Abcam) for Herceptin. Plates were washed and developed using standard protocols. FcγR-binding assays were performed as described elsewhere^33^, except that 50 μl of 5 μg ml^−1^ of cyclonals with the WT and mutant Fc domain (cyclonal^Fc(E382V/M428I)^) both purified from SHuffle cells and WT IgG purified from hybridoma cells were diluted in 0.05 M Na_2_CO_3_ (pH 9.6) buffer and used to coat 96-well polystyrene ELISA plates overnight at 4 °C. Following blocking, the plate was incubated with serially diluted recombinant human FcγRI/CD64 (R&D Systems) at room temperature for 1 h. After washing, 1:20,000-diluted rabbit anti-6 × -His-tag antibody–HRP conjugate (Abcam) was added and plates were washed and developed using standard protocols.

### Gel filtration

Protein-A-purified cyclonals were analysed by size-exclusion chromatography using a Superdex 200 10/300 GL gel filtration column and the AKTA Purifier FPLC system. The Superdex 200 was equilibrated with 60 ml (∼2 CV) of PBS gel filtration buffer (50 mM phosphate buffer, 150 mM NaCl, pH 7.0) at a flow rate of 0.5 ml min^−1^. A total of 300 μl of protein-A-purified sample concentrated to 1 mg ml^−1^ was loaded onto and passed through the column at 0.25 ml min^−1^ with gel filtration buffer. Absorbance at 280 nm (Abs_280_) was measured and recorded using UNICORN 5.1 software. Apparent molecular weights were calculated by interpolation using the elution volume at the maxima of each major absorption peak and a calibration curve generated using gel filtration high-molecular weight standards (GE healthcare).

### Serum stability

Protein-A-purified IgGs from SHuffle or hybridoma cell culture were diluted to a final concentration of 30 μg ml^−1^ in 100% fetal bovine serum (Sigma) and incubated at 37 °C for 4 days. Residual binding activity to MBP was evaluated by ELISA as described above.

### Surface plasmon resonance

Equilibrium binding-affinity measurements were made using a Biacore 3000 system. Cyclonal IgG or scFv antibodies were covalently linked to the surface of a CM5 sensor chip through amine coupling chemistry. First, the surface of the CM5 sensor chip was activated using *N*-hydroxysuccinimide and *N*-ethyl-*N*-(3-dimethylaminopropyl)carbodiimide hydrochloride (GE Healthcare). Purified anti-Gcn4 cyclonals or scFvs diluted to 50 μg ml^−1^ in 10 mM sodium acetate pH 4.0 were immobilized to the surface of the CM5 chip with a target level of 900 response units (RUs). Unreacted sites on the chip surface were subsequently blocked with 1 M ethanolamine hydrochloride. Serial dilutions of the antigen, MBP–TEV–Gcn4, prepared in 10 mM HEPES, pH 7.4, 150 mM NaCl, 3 mM EDTA, 0.005% polysorbate-20 (HBS-EP buffer, GE Healthcare) at concentrations ranging from 0.5 to 32 nM were injected over the chip using the same buffer at a flow rate of 30 μl min^−1^ (2 min injection time, 5 min dissociation). The surface of the chip was regenerated between the injections of each serial dilution with 10 mM glycine, pH 2.0 (2 min injection time, 2 min stabilization time). Kinetic parameters (*k*_a_, *k*_d_ and *K*_D_) were determined by fitting the response curves with a Langmuir 1:1 binding model within the BIAevaluation software.

## Additional information

**How to cite this article:** Robinson, M.-P. *et al.* Efficient expression of full-length antibodies in the cytoplasm of engineered bacteria. *Nat. Commun.* 6:8072 doi: 10.1038/ncomms9072 (2015).

## Supplementary Material

Supplementary Information Supplementary Figures 1-7, Supplementary Tables 1-4 and Supplementary References
