# Supplementary material for: Efficient expression of full-length antibodies in the cytoplasm of engineered bacteria
Source: Nat Commun. 2015 Aug 27;6:8072. doi: 10.1038/ncomms9072 (PMC4560801; doi:10.1038/ncomms9072)
Supplement: Supplementary Information — Supplementary Figures 1-7, Supplementary Tables 1-4 and Supplementary References [file ncomms9072-s1.pdf]

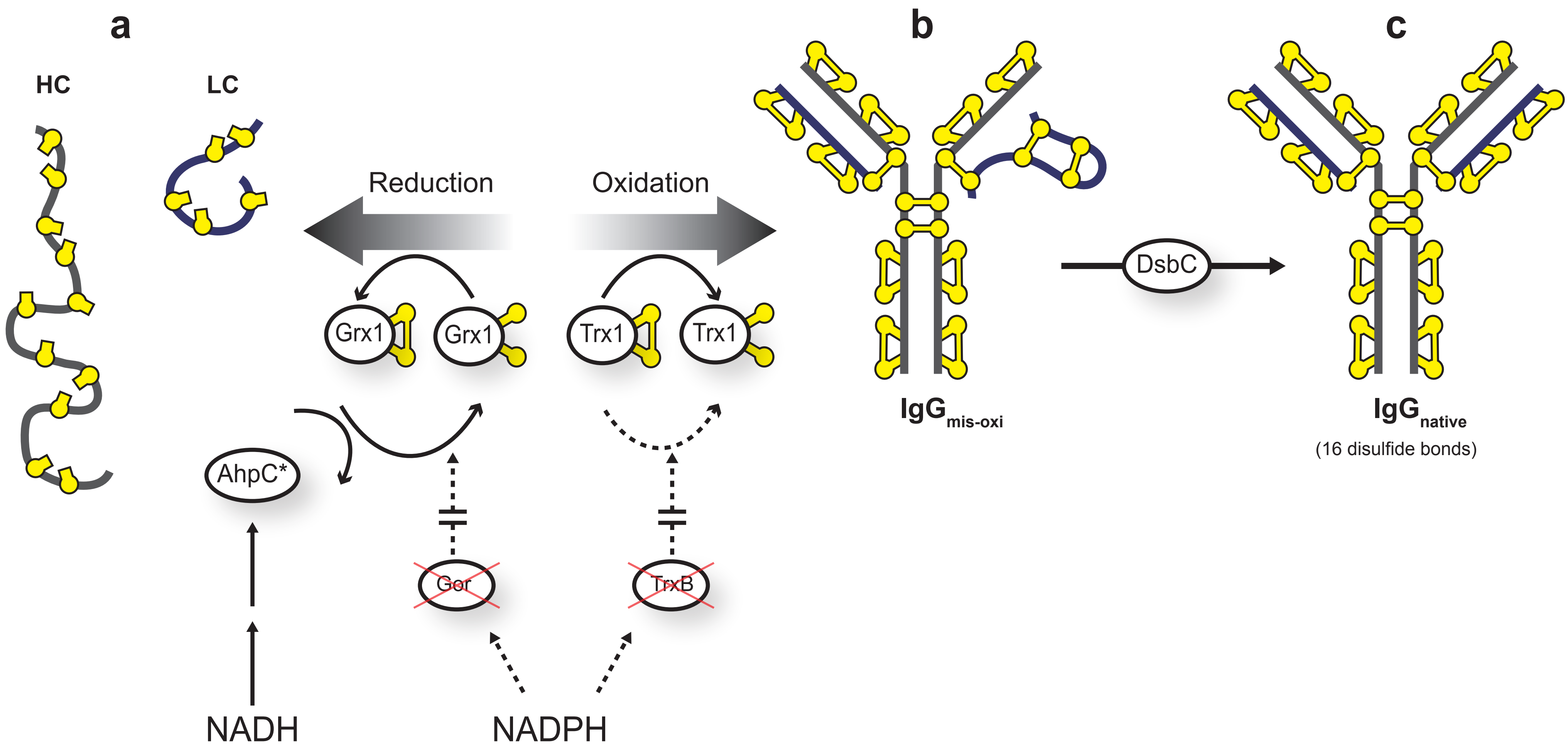

Supplementary Figure 1. Disulfide bond formation in the cytoplasm of SHuffle. Schematic diagram of the redox pathways in the cytoplasm of SHuffle cells. Disabled protein interactions due to the deletion of *trxB* and *gor* are represented as dotted lines. Redox state of cysteines (yellow balls) are indicated (oxidized = ball + stick; reduced = ball). (a) Heavy chain (HC) with 11 cysteines and light chain (LC) with 5 cysteines are reduced by Grx1 or oxidized by Trx1. (b) Mis-oxidized IgG is isomerized to its (c) native correctly folded state by DsbC.

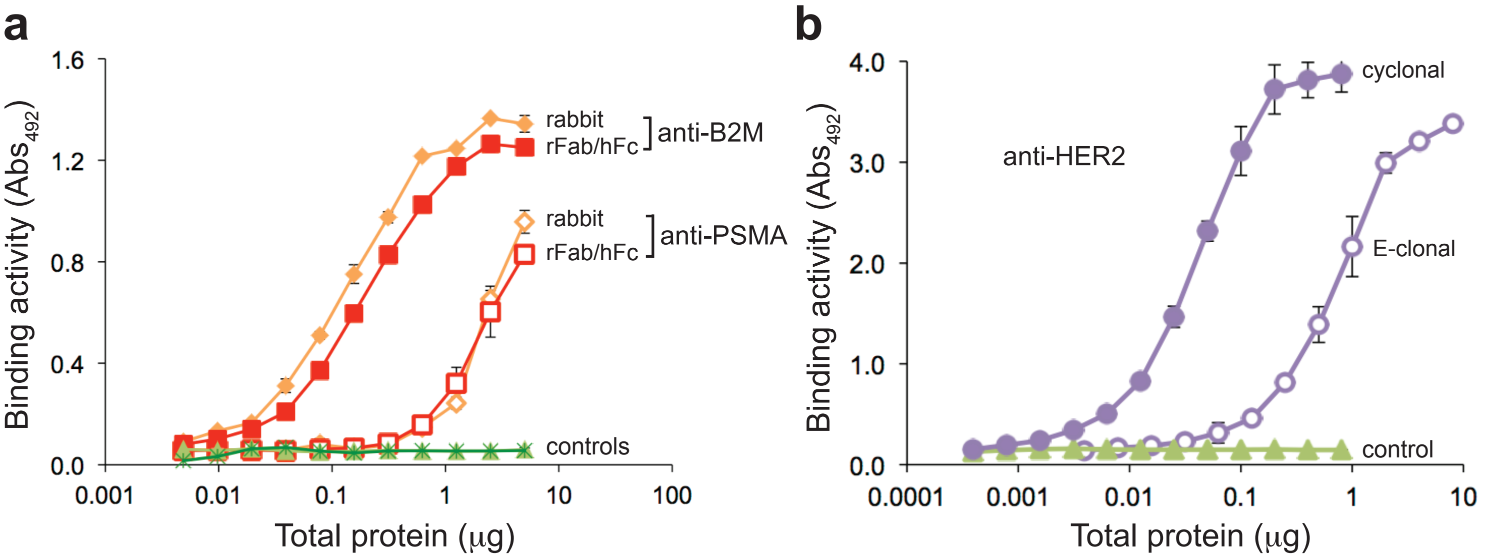

Supplementary Figure 2. Antigen-binding activity of rabbit and humanized cyclonals. (a) Antigen-binding activity in SHuffle lysates for anti-β2 microglobulin (B2M) and anti-prostate-specific membrane antigen (PSMA) cyclonals as indicated. ELISA signals (Abs_{492}) for rabbit cyclonals (diamonds) and empty plasmid control (triangles) were obtained using anti-rabbit antibodies; signals for rFab/hFc cyclonals (squares) and corresponding empty plasmid control (asterisks) were obtained with anti-human Fc antibodies. (b) HER2-binding activity in lysates for cyclonal (closed circles) and E-clonal (open circles) versions of Herceptin expressed from pMAZ360-Herceptin in SHuffle cells or from pSTJ4-AglycoT in the parental B strain, as well as for empty plasmid control (triangles). ELISA signals (Abs_{492}) were obtained with anti-human HC+LC antibodies. All data is expressed as the mean ± standard error of the mean (SEM) of biological triplicates.

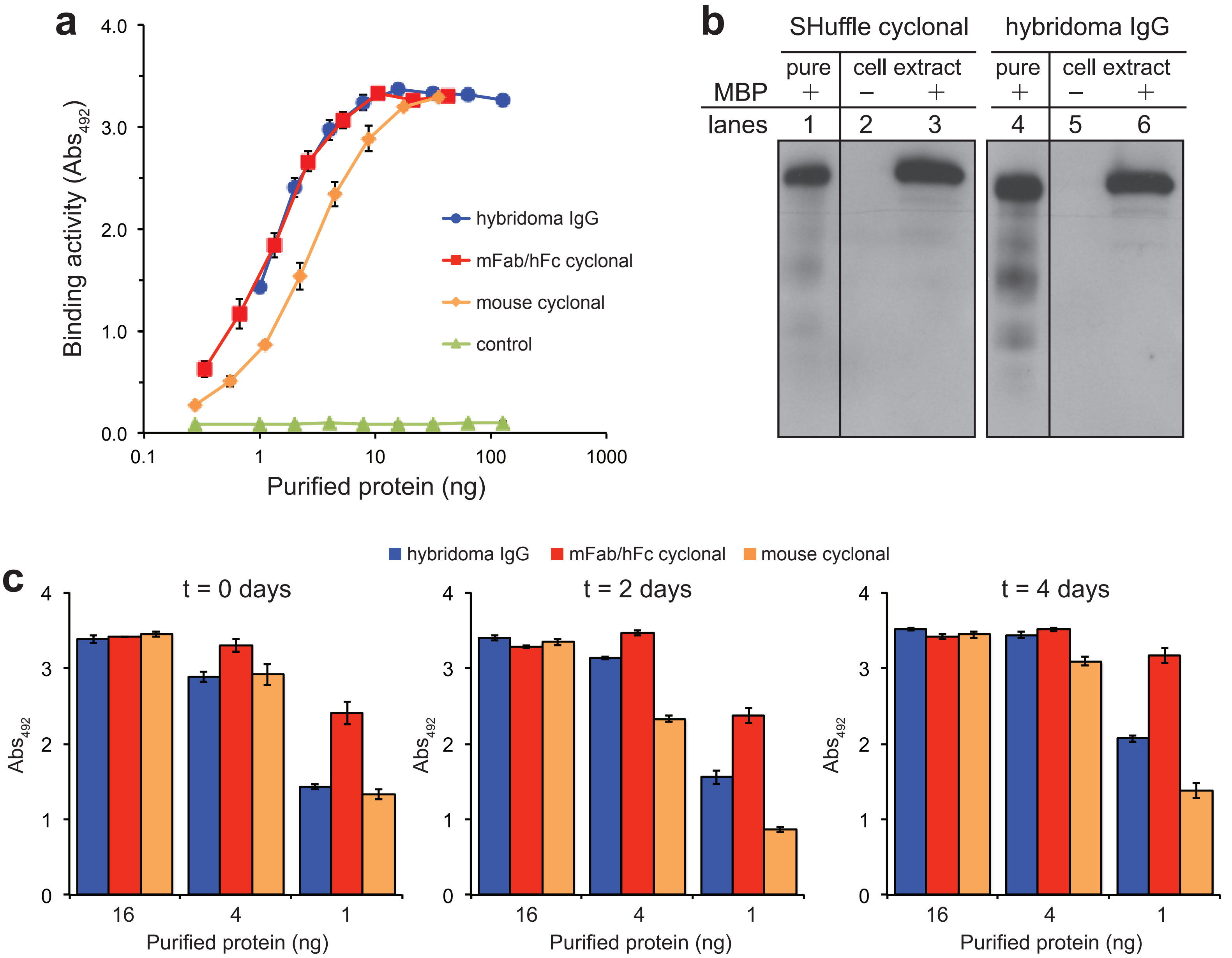

Supplementary Figure 3. Comparison of SHuffle- versus hybridoma-derived IgGs. (a) Antigen-binding activity for protein A-purified hybridoma-produced anti-MBP monoclonal IgGs or anti-MBP cyclonals in the mFab/hFc hybrid or mouse formats. ELISA signals (Abs_{492}) for all samples were obtained with anti-mouse IgG (Fab specific)-HRP antibodies. Data is expressed as the mean ± SEM of biological triplicates. (b) Western blots against purified MBP (lane 1 and 4) or MBP in crude cell lysates (lane 3 and 6) were probed with equal amounts of either SHuffle-produced anti-MBP cyclonals (lanes 1-3) or hybridoma-produced anti-MBP monoclonal IgGs (lanes 4-6). Cell lysates lacking cytoplasmic MBP served as controls and showed no unspecific cross-reaction for either of the IgGs (lanes 2 and 5). Each blot was incubated with 10-ml solution containing each antibody at a final concentration of 0.1 µg/ml. (c) Stability analysis for protein A-purified hybridoma-produced anti-MBP monoclonal IgGs or anti-MBP cyclonals in the mFab/hFc hybrid or mouse formats upon incubation in bovine serum. IgGs were diluted to a final concentration of 30 µg/ml in 100% bovine serum and incubated at 37°C for the indicated time periods. Residual binding activity to MBP of each fraction was evaluated by ELISA.

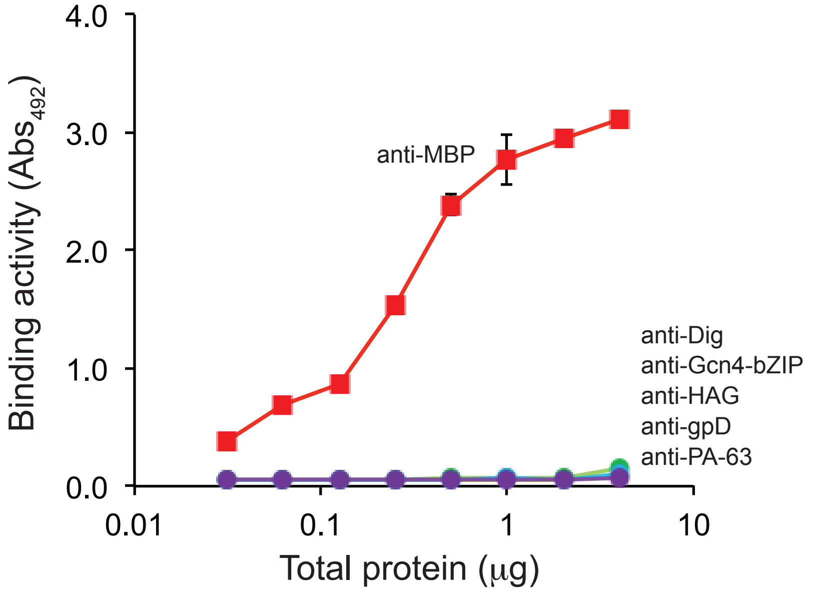

Supplementary Figure 4. Recognition of MBP by anti-MBP cyclonal and grafted derivatives. MBP-binding activity in SHuffle lysates for anti-MBP cyclonal and derivatives containing swapped V_H and V_L domains with specificity for antigens as indicated. ELISA signals (Abs_{492}) for the parental anti-MBP cyclonal in the mFab/hFc format (red) and all derivatives in the mFab/hFc format (various colors) were obtained with anti-human Fc antibodies. Data is the mean ± SEM of biological triplicates.

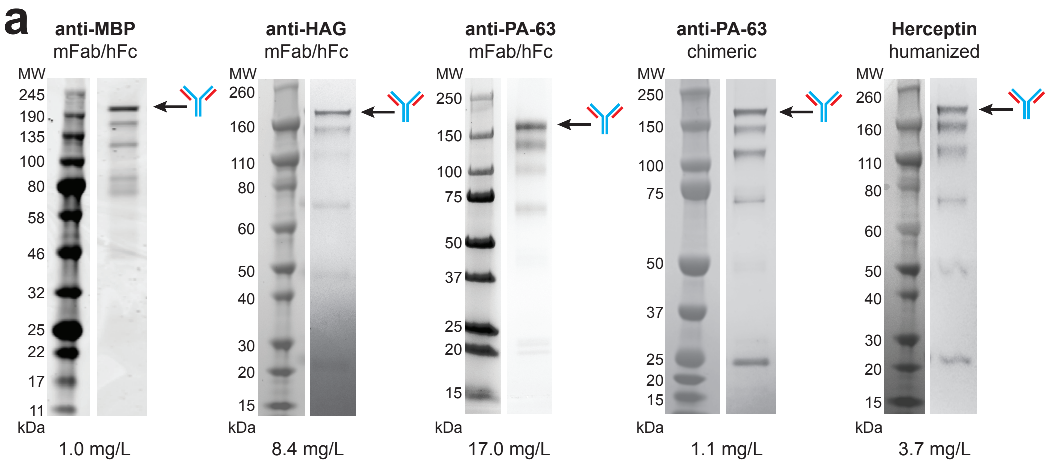

b

Cyclonal	Format	Plasmid	Titer (mg/L)
anti-MBP*	mFab/hFc	pET21b	1.0
anti-MBP	mFab/hFc	pMAZ360	3.6
anti-digoxin	mFab/hFc	pMAZ360	7.6
anti-HAG*	mFab/hFc	pMAZ360	8.4
anti-Gcn4-bZIP	mFab/hFc	pMAZ360	21.4
anti-gpD	mFab/hFc	pMAZ360	6.8
anti-PA-63*	mFab/hFc	pMAZ360	17.0
anti-PA-63*	chimeric	pMAZ360	1.1
Herceptin*	humanized	pMAZ360	3.7

*shown in SDS-PAGE above (Supplementary Fig. S5a)

Supplementary Figure 5. Purification of cyclonals from the cytoplasm of SHuffle cells. (a) Representative non-reducing SDS-PAGE gels of cyclonals purified from SHuffle T7 cells. Expression of each cyclonal was induced with 1 mM IPTG for 16 h at 30°C. Cyclonals were purified from cell lysate by affinity chromatography involving protein A columns. Molecular weight (MW) markers are shown at left. Arrows indicate fully assembled cyclonal IgG antibodies. (b) Representative titers for different cyclonals following protein-A purification strategy described in (a).

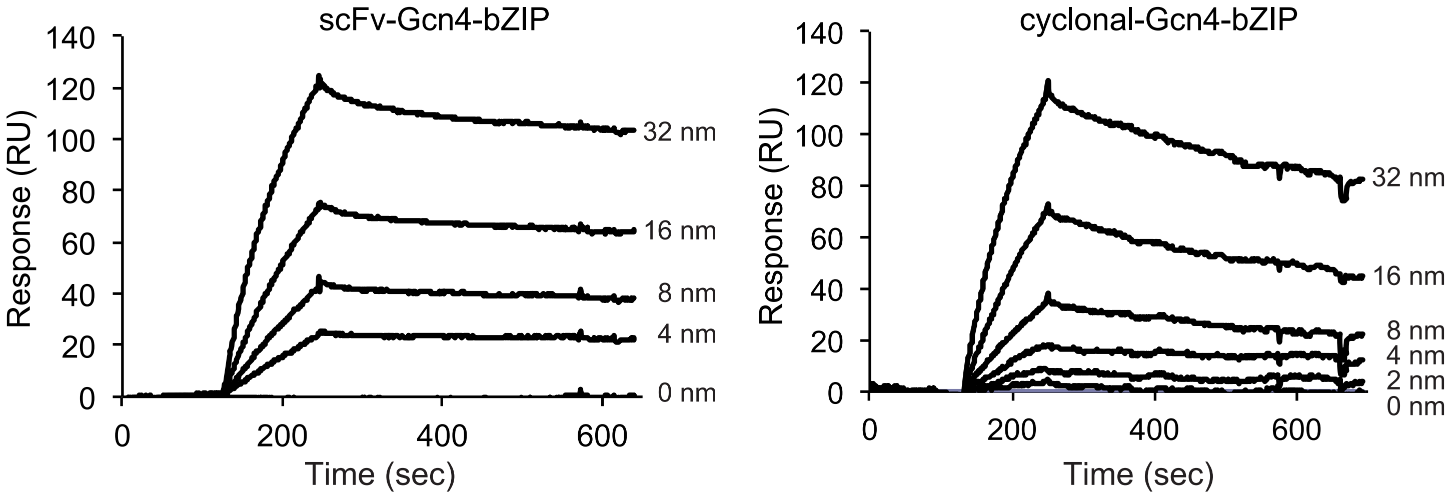

Clone	antigen	k_a (M^{-1} s^{-1})	k_d (s^{-1})	K_D (M)	Chi^2
scFv-Gcn4-bZIP	MBP-Gcn4	4.9×10^5	2.6×10^{-4}	$5.3 \pm 0.1 \times 10^{-10}$	1.1
cyclonal-Gcn4-bZIP	MBP-Gcn4	1.4×10^5	7.9×10^{-4}	$5.5 \pm 0.1 \times 10^{-9}$	2.8

Supplementary Figure 6. Binding kinetics of cyclonal versus scFv antibodies. Biacore sensorgrams generated for purified scFv-Gcn4-bZIP (left) and cyclonal-Gcn4-bZIP (right) measured by surface plasmon resonance. Binding kinetics were monitored using Biacore 3000. Purified scFv-Gcn4-bZIP or cyclonal-Gcn4-bZIP was immobilized on CM5 chips and the response of varied concentrations of purified MBP-Gcn4, given in each curve, was compared with an empty flow cell. Purity of all recombinant proteins was assessed by SDS-PAGE. Affinity values were obtained by fitting the equilibrium binding responses with a 1:1 Langmuir binding model using a simultaneous non-linear program. Representative results are depicted.

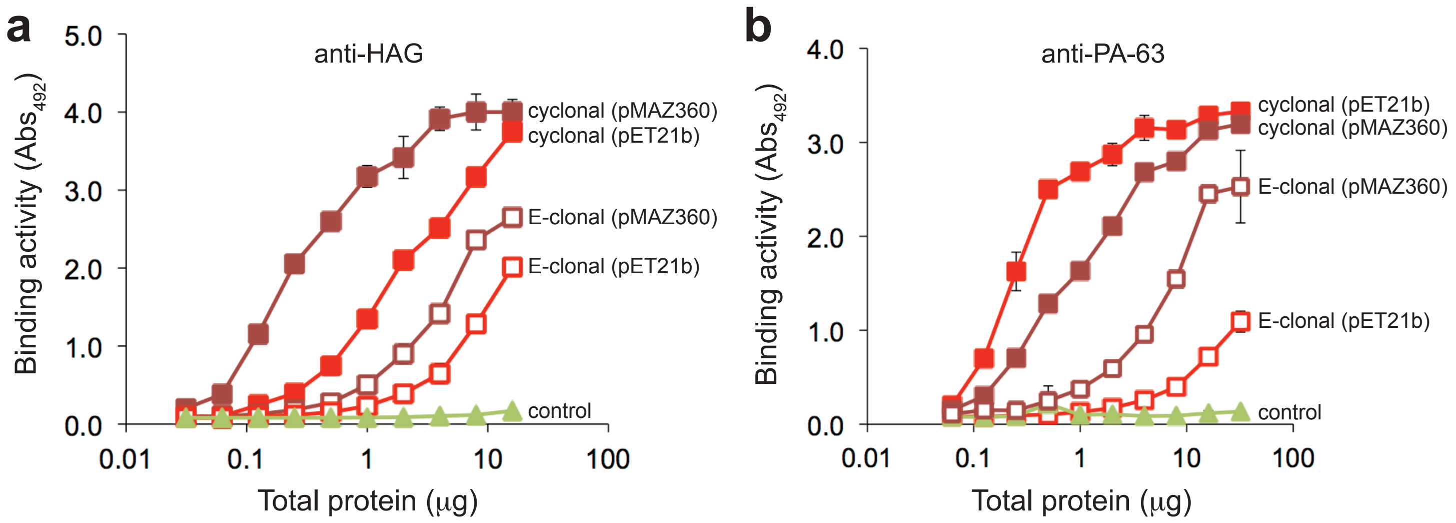

Supplementary Figure 7. Cytoplasmic versus periplasmic IgG expression. Antigen-binding activity of mFab/hFc IgGs specific for (a) HAG or (b) PA-63. Cyclonals (filled squares) were produced in the cytoplasm of K12 strain SMG96; E-clonals (open squares) were produced in the periplasm of the parental K12 strain. Expression was either from plasmid pET21b (red squares) or pMAZ360 (dark red squares); signal from cells carrying empty plasmid was used as negative control (green). Data is expressed as the mean ± SEM of biological triplicates.

Supplementary Table 1. Effects of helper protein co-expression on cyclonal production

Description	Helper	Normalized activity[1]
Redox active helpers	pBAD33	1.0
	AaDsbC	**1.4**
	DsbC	1.2
	$TrxA_{CGPC}$	1.1
	$TrxA_{CPYC}$	**1.3**
	$TrxA_{CPHC}$	**1.5**
	AaPDO	1.2
	QSOX	1.2
	PDI	1.2
	EUG1	1.1
	MPD1	**1.7**
	MPD2	1.2
Chaperone helpers	MalE	1.2
	Skp	0.9
Oxidative stress helpers	KatG	**1.4**
	AhpC, AhpF	1.1
	AhpC*, AhpF	0.9

[1]Activity of anti-MBP cyclonal in crude cell lysates derived from SHuffle cells was quantified by ELISA. All samples were normalized according to total protein as determined by Bradford assay and the activity data was normalized to the activity for cells expressing empty vector alone (pBAD33). Bold font indicates the five best helpers in terms of fold improvement.

Supplementary Table 2. Strains and plasmids used in this study

Strain or plasmid	Relevant genotype or features	Source
Strain		
NEB express T7	BL21 *fhuA2 lacZ::T7 gene1 [lon] ompT gal sulA11 R*(*mcr-73::miniTn10*--Tet^S)*2 [dcm] R*(*zgb-210::Tn10*--Tet^S) *endA1* Δ(*mcrC-mrr*)*114::IS10*	NEB
MB1731	NEB express T7 Δ*trxB* Δ*gor ahpC**	(1)
SHuffle T7 express	MB1731 λ*att::pNEB3-r1-cDsbC* ($Spec^R$, *lacI*^{q})	NEB
Plasmid		
pBAD33-cAaPDO	Gene encoding *Aquifex aeolicus* oxido-reductase (PDO) in plasmid pBAD33	(1)
pBAD34-cAaDsbC	Gene encoding *A. aeolicus* DsbC homolog devoid of signal peptide in plasmid pBAD34	(1)
pBAD33-cDsbC	Gene encoding *E. coli* DsbC devoid of signal peptide in plasmid pBAD33	(1)
pBAD33-$TrxA_{CGPC}$	Gene encoding *E. coli* thioredoxin A (TrxA) with wt active site (CGPC) in plasmid pBAD33	(1)
pBAD33-$TrxA_{CPYC}$	Gene encoding *E. coli* TrxA with *grx* active site (CPYC) in plasmid pBAD33	(1)
pBAD33-$TrxA_{CPHC}$	Gene encoding *E. coli* TrxA with *dsbA* active site (CPHC) in plasmid pBAD33	(1)
pBAD33-QSOX	Gene encoding human quiescin sulphydryl oxidase in plasmid pBAD33	(1)
pBAD34-MalE	Gene encoding *E. coli* maltose binding protein (MBP) devoid of signal peptide in plasmid pBAD34	(1)
pBAD34-PDI	Gene encoding yeast protein disulfide isomerase (PDI) in plasmid pBAD34	(1)
pBAD34-EUG1	Gene encoding yeast PDI homolog (EUG1) in plasmid pBAD34	(1)
pBAD34-MPD1	Gene encoding yeast PDI homolog (MPD1) in plasmid pBAD34	(1)
pBAD34-MPD2	Gene encoding yeast PDI homolog (MPD2) in plasmid pBAD34	(1)
pBAD34-KatG	Gene encoding *E. coli* catalase (KatG) in plasmid pBAD34	(1)
pBAD33-AhpCF	Genes encoding *E. coli* wt peroxidase pair (AhpC and AhpF) in plasmid pBAD33	(1)
pBAD33-AhpC*F	Genes encoding *E. coli* mutant peroxidase pair (AhpC* and AhpF) in plasmid pBAD33	(1)
pBAD34-Skp	Gene encoding *E. coli* periplasmic chaperone Skp devoid of signal peptide in plasmid pBAD34	(1)
pMAZ360-IgG	Bicistronic expression of IgGs in the *E. coli* periplasm; HC and LC each carries N-terminal PelB signal peptide	(2)

pMAZ360-26.10-IgG	Bicistronic expression of 26.10-IgG in the *E. coli* periplasm	(2)
pMAZ360-YMF10-IgG	Bicistronic expression of YMF10-IgG in the *E. coli* periplasm	(2)
pHK19	Gene encoding Gcn4-specific scFv antibody	(3)
pHK38	Gene encoding HAG-specific scFv antibody	(4)
pHK49	Gene encoding gpD-specific scFv antibody	(5)
pET21b-cyclonal-MBP	Mouse anti-MBP HC and LC genes lacking export signals in pET21b; bicistronic expression	This study*
pET21b-cyclonal-MBP(mFab/hFc)	Mouse anti-MBP Fab spliced onto human Fc in pET21b; bicistronic expression	This study*
pET21b-cyclonal-MBP(chimeric)	Mouse anti-MBP variable domains fused to human constant domains (mouse V_L to human C_L and mouse V_H to human C_H1-C_H2-C_H3 for light and heavy chains, respectively) in pET21b; bicistronic expression	This study*
pET21b-cyclonal-Dig(mFab/hFc)	pET21b-cyclonal-MBP(mFab/hFc) but with V_H and V_L from murine 26-10-IgG that binds digoxin(6)	This study*
pET21b-cyclonal-Dig(chimeric)	pET21b-cyclonal-MBP(chimeric) but with V_H and V_L from murine 26-10-IgG that binds digoxin(6)	This study*
pET21b-cyclonal-Gcn4(mFab/hFc)	pET21b-cyclonal-MBP(mFab/hFc) but with V_H and V_L from anti-GCN4 scFv that binds Gcn4-bZIP domain(3)	This study*
pET21b-cyclonal-Gcn4(chimeric)	pET21b-cyclonal-MBP(chimeric) but with V_H and V_L from anti-GCN4 scFv that binds Gcn4-bZIP domain(3)	This study*
pET21b-cyclonal-gpD(mFab/hFc)	pET21b-cyclonal-MBP(mFab/hFc) but with V_H and V_L from D10 scFv that binds bacteriophage lambda gpD(5)	This study*
pET21b-cyclonal-gpD(chimeric)	pET21b-cyclonal-MBP(chimeric) but with V_H and V_L from D10 scFv that binds bacteriophage lambda gpD(5)	This study*
pET21b-cyclonal-HAG(mFab/hFc)	pET21b-cyclonal-MBP(mFab/hFc) but with V_H and V_L from the h6-4 scFv that binds 6-residue HAG peptide(4)	This study*
pET21b-cyclonal-HAG(chimeric)	pET21b-cyclonal-MBP(chimeric) but with V_H and V_L from the h6-4 scFv that binds 6-residue HAG peptide(4)	This study*
pET21b-cyclonal-PA-63(mFab/hFc)	pET21b-cyclonal-MBP(mFab/hFc) but with V_H and V_L from M18.1 Hum-IgG that binds *B. anthracis* PA-63(2)	This study*
pET21b-cyclonal-PA-63(chimeric)	pET21b-cyclonal-MBP(chimeric) but with V_H and V_L from M18.1 Hum-IgG that binds *B. anthracis* PA-63(2)	This study*

pET21b-cyclonal-PA63(Fc$^{E382V/M428I}$)	pET21b-cyclonal-PA-63(chimeric) but with E382V and M482I mutations in Fc domain	This study*
pET21b-E-clonal-MBP(mFab/hFc)	Mouse Fab/human Fc anti-MBP IgG in pET21b; PelB signal peptides on heavy and light chains	This study*
pMAZ360-cyclonal-MBP(mFab/hFc)	Mouse Fab/human Fc anti-MBP cyclonal in pMAZ360-IgG	This study*
pMAZ360-E-clonal-MBP(mFab/hFc)	Mouse Fab/human Fc anti-MBP IgG in pMAZ360-IgG; PelB signal peptides on heavy and light chains	This study*
pET21b-E-clonal-HAG(mFab/hFc)	Mouse Fab/human Fc anti-HAG IgG in pET21b; PelB signal peptides on heavy and light chains	This study*
pMAZ360-cyclonal-HAG(mFab/hFc)	Mouse Fab/human Fc anti-HAG cyclonal in pMAZ360-IgG	This study*
pMAZ360-E-clonal-HAG(mFab/hFc)	Mouse Fab/human Fc anti-HAG IgG in pMAZ360-IgG; PelB signal peptides on heavy and light chains	This study*
pET21b-E-clonal-PA-63(mFab/hFc)	Mouse Fab/human Fc anti-PA-63 IgG in pET21b; PelB signal peptides on heavy and light chains	This study*
pMAZ360-cyclonal-PA-63(mFab/hFc)	Mouse Fab/human Fc anti-PA-63 cyclonal in pMAZ360-IgG	This study*
pMAZ360-E-clonal-PA-63(mFab/hFc)	Mouse Fab/human Fc anti-PA-63 IgG in pMAZ360-IgG; PelB signal peptides on heavy and light chains	This study*
pSTJ4-AglycoT	Humanized anti-HER2/neu IgG in pMAZ360-IgG; PelB signal peptides on heavy and light chains	(7)
pMAZ360-Herceptin	Humanized anti-HER2/neu cyclonal in pMAZ360-IgG	This study
pET28a-gpD	gpD cloned in pET pET28a(+), which introduced a 6x-His tag to the N-terminus	This study
pET28a-GST-Gcn4	Gcn4-bZIP domain cloned as GST fusion in pET pET28a(+), which introduced N-terminal 6x-His tag	This study
pET28a-GST-HAG	6-residue HAG peptide cloned as GST fusion in pET pET28a(+), which introduced N-terminal 6x-His tag	This study

*Construction of plasmids described in Supplementary Table 3.

Supplementary Table 3. Primers used in construction of plasmids.

Name	Sequence (5’ to 3’)
50	TATATATATATATATATTCTAGAGAAGGAGATATACACATGCAGGTCCAACTGCAGCAACCTGGG
51	TATGCGGCGCGCCTTATTATTTACCCGGAGTCCGGGAGAAGC
52	ATATACATATGGACATTGTGATGACACAGTCTCC
53	GTGTATATCTCCTTCTCTAGAATATATATATATATATATTAACACTCATTCCTGTTGAAGC
123	ATATACATATGGACATTGTGATGAC
124	ATGCCTCGAGTTATTTACCCGGAGTCCGGGAGAAGCTC
160	ATATACATATGGACATTGTGATGAC
161	CCGTACGTTTCATGTCGACTTTGGTCCCCCCCCCGAACGTGTACGGAG
162	CTCCGTACACGTTCGGGGGGGGGACCAAAGTCGACATGAAACGTACGG
163	GGTTGCTGCAGTTGGACCTGCATGGCTAGCTCTCCTTCTCTAGAATA
164	TATTCTAGAGAAGGAGAGCTAGCCATGCAGGTCCAACTGCAGCAACC
165	GGTACCCTGGCCCCAGTATTTAAATAACCCTCTTGTACAGTAATAGACCGCAGA
166	TCTGCGGTCTATTACTGTACAAGAGGGTTATTTAAATACTGGGGCCAGGGTACC
167	ACGGTGGGCATGTGTGAGTTTTGTCGGCGCGCCCTTTCGGTTCAACTTTT
NK397	GCCGAGCTCGAATTCGTTAACAGTCACCCCTATTGAAGC
NK399	CGCGTGGCCGGCCGATTTATTTACCCGGAGAGCG
NK400	GTTTTGTCGGCGCGCCCGCATGTCGAGGGCG
NK401	TGCGCCCTCGACATGCGGGCGCGCCGA
NK402	CGCGTGGCCGGCCGATTTATTTACCCGGAGACAGGG
NK438	CTTTAAGAAGGAGATATACATGGCCGTGCTGACCC
NK439	GTATAAGAAGGAGATATACAATGCAGTCGGTGGAGGAGTCC
NK440	GTATAAGAAGGAGATATACAATGCAGTCGCTGGAGGAGTCC
Deg1	GGGAATTCCACCATGGASACAGACACACTCCTGCTATGG
Deg2	GGGAATTCCACCATGGATTTTCWRGTGCAGATYTTCAG
Deg3	GGGAATTCCACCATGRAKTCACAKRCYCAGGTYYTYAT

Deg4	GGGAATTCCACCATGAKKNHYTCWSCTCAGYTYYTKGG
Deg5	GGGAATTCCACCATGAAGTTGCCTGTTAGGCTGTTG
Deg6	GGGAATTCCACCATGGACATRAGKRCYCYYGCTCAG
Deg7	GGGAATTCCACCATGCATCAGAYCAGCATGGGCWTCAAG
LC-9	GCGCCGGTCGACATTAACACTCATTCCTGTTGAAGC
Kabat-F	GGGAATTCCACCATGGRATGSAGCTGKGTMATSCTC
Kabat-R	GTGGGGCGGCCGCTCATTTACCCGGAGTCCGGGAGAAGC
201	TTTTTTCATATGGCGGACATAGTACTGACCCAGTC
202	GATGGATACAGTTGGTGCAGCATCAGTGGCCGATTTGATCTCGAGCTTG
203	CAAGCTCGAGATCAAATCGGCCACTGATGCTGCACCAACTGTATCCATC
204	TTTTTTGCTAGCCATGGCGGAGCTGCAGCAGTC
205	CCGATGGGGCTGTTGTTTTGGCAGAGCTCACAGTAACACTAGCACCATG
206	CATGGTGCTAGTGTTACTGTGAGCTCTGCCAAAACAACAGCCCCATCGG
207	TTTTTTCATATGCGAGATATCGTTATGACCCAATCTC
208	GATACAGTTGGTGCAGCATCAGCGCGCTTAAGCTCCACTTTGGTTC
209	GAACCAAAGTGGAGCTTAAGCGCGCTGATGCTGCACCAACTGTATC
210	TTTTTTGCTAGCCATGGAAGTCAAACTTCTTGAGTCAGGTG
211	GTTGTTTTGGCTGCAGAGACAGTAACCAGCGTGCCCTGC
212	GCAGGGCACGCTGGTTACTGTCTCTGCAGCCAAAACAAC
213	TTAAATCATATGGATATCGAACTGACCCAGCCG
214	CCCGTTTTATTTCCAGCTTGGTGCCGCCGCCAAACACAG
215	CTGTGTTTGGCGGCGGCACCAAGCTGGAAATAAAACGGG
216	TTTTTTAAGCTTTTAACACTCATTCCTGTTGAAGCTC
217	ATTAATGCTAGCCATGGAAGTGCAATTGGTGGAAAGCG
218	GATGGGGCTGTTGTTTTGGCTGCGCTAACCGTCACCAGGGTGC
219	GCACCCTGGTGACGGTTAGCGCAGCCAAAACAACAGCCCCATC
220	GTGGGCATGTGTGAGTTTTGTC
221	AAAAAACATATGGATATCGTTATGACCCAGTCACCG

222	GATGGATACAGTTGGTGCAGCATCAGCGCGCTTAAGTTCCAGTTTG
223	CAAACTGGAACTTAAGCGCGCTGATGCTGCACCAACTGTATCCATC
224	ATTAATGCTAGCCATGGAAGTTCAACTAGTTGAATCCGGTGGTG
225	CGATGGGGCTGTTGTTTTGGCAGCTGAAACGGTAACCAGGGTAC
226	GTACCCTGGTTACCGTTTCAGCTGCCAAAACAACAGCCCCATCG
227	TTTTTTCATATGGCGGATATTGTGATGACCCAGTC
228	GATGGATACAGTTGGTGCAGCATCAGTACGATTCAGCTCCAGCTTGG
229	CCAAGCTGGAGCTGAATCGTACTGATGCTGCACCAACTGTATCCATC
230	TTTTTTGCTAGCCATGGCGGAGGTCCAGCTTC
231	CCGATGGGGCTGTTGTTTTGGCTGAGGAGACTGTGAGAGTGGTG
232	CACCACTCTCACAGTCTCCTCAGCCAAAACAACAGCCCCATCGG
233	TTTTTTGTCGACCTTGGTCCCCGAGCCGAAC
234	CACCAGGGTACCCTGGCCCCAATAATCCATGGCCCATTTGTTACCAG
235	CTGGTAACAAATGGGCCATGGATTATTGGGGCCAGGGTACCCTGGTG
236	TTTTTTGTCGACTTTGGTTCCTTGACCAAATACCCAGTG
237	CAGGGTACCCTGGCCCCAGTAGTCGAAAAGTCCTGTCACGCAG
238	CTGCGTGACAGGACTTTTCGACTACTGGGGCCAGGGTACCCTG
239	TTTTTTGTCGACCTTCGTGCCGCCGCCAAACAC
240	CACCAGGGTACCCTGGCCCCAATAATCCATACCAGAAACATAAGAAAAACGC
241	GCGTTTTTCTTATGTTTCTGGTATGGATTATTGGGGCCAGGGTACCCTGGTG
242	TTTTTTGTCGACTTTGGTGCCACCACCGAAGGTC
243	CAGGGTACCCTGGCCCCAGTAGGCGAAACCGTTTTCGTCATAAC
244	GTTATGACGAAAACGGTTTCGCCTACTGGGGCCAGGGTACCCTG
245	TATATAGGCGCGCCCGCAGCTTTTCGGTTCAACTTTTTATCCACTTTCG
246	CTGCCCATTGCTCACCCACTCCACGGC
247	GCCGTGGAGTGGGTGAGCAATGGGCAG
248	GAGCCTCGTGGATCACGGAGC
249	GAGAGGCTCTTCTGCGTGTAGTGGTTGTGCAGAGCCTCGTGGATCACGGAGCATGAGAAG

250	CTCAGC CTCGAG TCATTTACCCGGAGACAGGGAGAGGCTCTTCTGCGTGTAGTGG
251	TGGATTGTTATTACTCGCGGCCCAGCCGGCCATGGCGGACATTGTGATGACACAGTCTCC
252	CTCAGT CATATG AAATACCTATTGCCTACGGCAGCCGCTGGATTGTTATTACTCGCGGC
253	CCGCTGGATTGTTATTGCTAGCGGCTCAGCCGGCAATGGCGCAGGTCCAACTGCAGCAAC
254	AGGAGAGAATTCCATGAAATACCTATTGCCTACGGCAGCCGCTGGATTGTTATTGCTAGC
255	CTCATG AAGCTT TATATATATATATATATTCTAGAGAAGGAGAGAATTCCATGAAATACCTATTG
256	CTCAGT CTCGAG TCATTTACCCGGAGAC
257	TTTTTTCCATGGCGGATATCGTTATGACCCAGTCACCGTC
258	GCAATGGCGGAAGTTCAACTAGTTGAATCCGGTGGTG
259	TATAATGCTAGCGGCTCAGCCGGCAATGGCGGAAGTTCAACTAGTTG
260	GATTCA CCATGG CGGATATTGTGATGACC
261	GAACTT AAGCTT TTAACACTCATTCCTGTTGAAGCTCTTGAC
262	CTCAGTTCTAGAATGAAATCCCTATTGCCTACGGC
263	AATATTACGCGTGTACTCGAGTTATCATTTACCCGGAGACAGGGAG
264	TATATT CATATGGACATTGTGATGACACAGTC
265	GACGGGCCTTTGGTGCTGGCTGAGGAGACTGTGAGAGTGGTG
266	CACCACTCTCACAGTCTCCTCAGCCAGCACCAAAGGCCCGTC
267	TTAATTGTCGACCTTGGTCCCAGCACCGAAC

*Engineered restriction sites are underlined.

Supplementary Table 4. Construction of plasmids used in this study.

Plasmid	Template	Primer pair	Restriction Enzymes
pMAZ-cyclonal-MBP	pNEB-LC-MBP<br>pUC57-HC-MBPsyn*<br>PCR (cLC + cHC)	52-53 (= PCR cLC)<br>50-51 (= PCR cHC)<br>52-51 (= PCR cLH)	PCR cLH *Nde*I/*Asc*I
pET21b-cyclonal-MBP	pMAZ-cyclonal-MBP	123-124	*Nde*I/*Xho*I
pET21b-cyclonal-MBP(mFab/hFc)	pUC57-mFab/hFcsyn*	*Asc*I/*Xho*I fragment	*Asc*I/*Xho*I
pET21b-cyclonal-MBP(chimeric)	pET21b-cyclonal-MBP(mFab/hFc)<br>pUC57-hLCsyn*<br>pET21b-cyclonal-MBP(mFab/hFc)<br>pUC57-hHCsyn*<br>PCR (A + B + C + D)	160-161 (= PCR A)<br>162-163 (= PCR B)<br>164-165 (= PCR C)<br>166-167 (= PCR D)<br>160-167 (= PCR E)	PCR E *Nde*I/*Asc*I
pET21b-cyclonal-Dig(mFab/hFc)	pMAZ360-26.10-IgG<br>pET21b-cyclonal-MBP(mFab/hFc)<br>pMAZ360-26.10-IgG<br>pET21b-cyclonal-MBP(mFab/hFc)<br>PCR (A+B)<br>PCR (C+D)	201-202 (= PCR A)<br>203-216 (= PCR B)<br>204-205 (= PCR C)<br>206-220 (= PCR D)<br>201-216 (= PCR E)<br>204-220 (= PCR F)	PCR E *Nde*I/*Hin*dIII<br>PCR F *Nhe*I/*Asc*I
pET21b-cyclonal-Gcn4(mFab/hFc)	pHK19 (anti-GCN4 scFv)<br>pET21b-cyclonal-MBP(mFab/hFc)<br>pHK19 (anti-GCN4 scFv)<br>pET21b-cyclonal-MBP(mFab/hFc)<br>PCR (A+B)<br>PCR (C+D)	207-208 (= PCR A)<br>209-216 (= PCR B)<br>210-211 (= PCR C)<br>212-220 (= PCR D)<br>207-216(= PCR E)<br>210-220 (= PCR F)	PCR E *Nde*I/*Hin*dIII<br>PCR F *Nhe*I/*Asc*I
pET21b-cyclonal-gpD(mFab/hFc)	pHK49 (anti-gpD scFv, D10)<br>pET21b-cyclonal-MBP(mFab/hFc)	213-214 (= PCR A)<br>215-216 (= PCR B)	

	pHK49 (anti-gpD scFv, D10)<br>pET21b-cyclonal-MBP(mFab/hFc)<br>PCR (A+B)<br>PCR (C+D)	217-218 (= PCR C)<br>219-220 (= PCR D)<br>213-216 (= PCR E)<br>217-220 (= PCR F)	 <br>PCR E *Nde*I/*Hin*dIII<br>PCR F *Nhe*I/*Asc*I
pET21b-cyclonal-HAG(mFab/hFc)	pHK38 (anti-Hag scFv)<br>pET21b-cyclonal-MBP(mFab/hFc)<br>pHK38 (anti-Hag scFv)<br>pET21b-cyclonal-MBP(mFab/hFc)<br>PCR (A+B)<br>PCR (C+D)	221-222 (= PCR A)<br>223-216 (= PCR B)<br>224-225 (= PCR C)<br>226-220 (= PCR D)<br>221-216 (= PCR E)<br>224-220 (= PCR F)	 <br><br><br>PCR E *Nde*I/*Hin*dIII<br>PCR F *Nhe*I/*Asc*I
pET21b-cyclonal-PA63(mFab/hFc)	pMAZ360-YMF10-IgG<br>pET21b-cyclonal-MBP(mFab/hFc)<br>pMAZ360-YMF10-IgG<br>pET21b-cyclonal-MBP(mFab/hFc)<br>PCR (A+B)<br>PCR (C+D)	227-228 (= PCR A)<br>229-216 (= PCR B)<br>230-231 (= PCR C)<br>232-220 (= PCR D)<br>227-216 (= PCR E)<br>230-220 (= PCR F)	 <br><br><br>PCR E *Nde*I/*Hin*dIII<br>PCR F *Nhe*I/*Asc*I
pET21b-cyclonal-Dig(chimeric)	pMAZ360-26.10-MBP<br>pMAZ360-26.10-MBP<br>pET21b-cyclonal-MBP(chimeric)<br>PCR (B+C)	201-233 (= PCR A)<br>204-234 (= PCR B)<br>235-245 (= PCR C)<br>204-245 (= PCR D)	PCR A *Nde*I/*Sal*I<br><br><br>PCR D *Nhe*I/*Asc*I
pET21b-cyclonal-Gcn4(chimeric)	pHK19 (anti-GCN4 scFv)<br>pHK19 (anti-GCN4 scFv)<br>pET21b-cyclonal-MBP(chimeric)<br>PCR (B+C)	207-236 (= PCR A)<br>210-237 (= PCR B)<br>238-245 (= PCR C)<br>210-245 (= PCR D)	PCR A *Nde*I/*Sal*I<br><br><br>PCR D *Nhe*I/*Asc*I
pET21b-cyclonal-gpD(chimeric)	pHK49 (anti-gpD scFv, D10)<br>pHK49 (anti-gpD scFv, D10)<br>pET21b-cyclonal-MBP(chimeric)<br>PCR (B+C)	213-239 (= PCR A)<br>217-240 (= PCR B)<br>241-245 (= PCR C)<br>217-245 (= PCR D)	PCR A *Nde*I/*Sal*I<br><br><br>PCR D *Nhe*I/*Asc*I

pET21b-cyclonal-HAG(chimeric)	pHK38 (anti-Hag scFv)<br>pHK38 (anti-Hag scFv)<br>pET21b-cyclonal-MBP(chimeric)<br>PCR (B+C)	221-242 (= PCR A)<br>224-243 (= PCR B)<br>244-245 (= PCR C)<br>224-245 (= PCR D)	PCR A *Nde*I/*Sal*I<br><br><br>PCR D *Nhe*I/*Asc*I
pET21b-cyclonal-PA63(chimeric)	pMAZ360-YMF10-IgG<br>pMAZ360-YMF10-IgG<br>pET21b-cyclonal-MBP(chimeric)<br>PCR (B+C)	227-267 (= PCR A)<br>230-265 (= PCR B)<br>266-245 (= PCR C)<br>230-245 (= PCR D)	PCR A *Nde*I/*Sal*I<br><br><br>PCR D *Nhe*I/*Asc*I
pET21b-cyclonal-PA63(Fc$^{E382V/M428I}$)	pET21b-cyclonal-PA63(chimeric)<br>pET21b-cyclonal-PA63(chimeric)<br>PCR B<br>PCR C<br>PCR (A+D)	230-246 (= PCR A)<br>247-248 (= PCR B)<br>247-249 (= PCR C)<br>247-250 (= PCR D)<br>230-250 (= PCR E)	 <br><br><br>PCR E *Nhe*I/*Xho*I
pMAZ360-cyclonal-MBP(mFab/hFc)	pET21b-cyclonal-MBP(mFab/hFc)	264-263	*Nde*I/*Mlu*I
pMAZ360-cyclonal-HAG(mFab/hFc)	pET21b-cyclonal-HAG(mFab/hFc)	221-263	*Nde*I/*Xho*I
pMAZ360-cyclonal-PA63(mFab/hFc)	pET21b-cyclonal-PA63(mFab/hFc)	227-263	*Nde*I/*Xho*I
pET21b-E-clonal-HAG(mFab/hFc)	pET21b-cyclonal-HAG(mFab/hFc)<br>PCR A	257-216 (= PCR A)<br>258-256 (= PCR B)<br>259-256 (= PCR C)	PCR A *Nco*I/*Hin*dIII<br><br>PCR C *Nhe*I/*Xho*I
pET21b-E-clonal-MBP(mFab/hFc)	pET21b-cyclonal-MBP(mFab/hFc)<br>PCR A<br>pET21b-cyclonal-MBP(mFab/hFc)<br>PCR C<br>PCR D	251-261 (= PCR A)<br>252-261 (= PCR B)<br>253-256 (= PCR C)<br>254-256 (= PCR D)<br>255-256 (= PCR E)	 PCR B *Nde*I/*Hin*dIII<br><br><br>PCR E HindIII/XhoI
pET21b-E-clonal-PA63(mFab/hFc)	pET21b-cyclonal-PA63(mFab/hFc)<br>pMAZ360-YMF10-IgG<br>pET21b-cyclonal-MBP(mFab/hFc)<br>PCR (B+C)	260-216 (= PCR A)<br>262-231 (= PCR B)<br>232-220 (= PCR C)<br>262-220 (= PCR D)	PCR A *Nco*I/*Hin*dIII<br><br><br>PCR D *Nhe*I/*Asc*I

pMAZ360-E-clonal-HAG(mFab/hFc)	pET21b-E-clonal-HAG(mFab/hFc)	262-263	*Nde*I/*Xho*I
pMAZ360-E-clonal-MBP(mFab/hFc)	pET21b-E-clonal-MBP(mFab/hFc)	263-263	*Nde*I/*Xho*I
pMAZ360-E-clonal-PA63(mFab/hFc)	pET21b-E-clonal-PA63(mFab/hFc)	262-263	*Nde*I/*Xho*I
pETDuet-cyclonal-PSMA	CST-rLC-PSMA<br>CST-rHC-PSMA	NK438-NK397<br>NK440-NK399	*Nco*I/*Bam*HI
pETDuet-cyclonal-B2M	CST-rLC-B2M<br>CST-rHC-B2M	NK438-NK397<br>NK440-NK399	*Nco*I/*Bam*HI
pETDuet-cyclonal-PSMA(rFab/hFc)	CST-rLC-PSMA<br>CST-rHC-PSMA<br>pUC57-mFab/hFcsyn*<br>PCR A + B	NK438-NK397<br>NK440-NK400 (= PCR-A)<br>NK401-NK402 (= PCR-B)<br>Gibson Assembly	*Nde*I/*Eco*RV
pETDuet-cyclonal-B2M(rFab/hFc)	CST-rLC-B2M<br>CST-rHC-B2M<br>pUC57-mFab/hFcsyn*<br>PCR A + B	NK438-NK397<br>NK439-NK400 (= PCR-A)<br>NK401-NK402 (= PCR-B)<br>Gibson Assembly	*Nde*I/*Eco*RV

*Gene synthesis, GenScript

References

1. Lobstein J*, et al.* (2012) SHuffle, a novel *Escherichia coli* protein expression strain capable of correctly folding disulfide bonded proteins in its cytoplasm. *Microb Cell Fact* 11:56.
2. Mazor Y, Van Blarcom T, Mabry R, Iverson BL, & Georgiou G (2007) Isolation of engineered, full-length antibodies from libraries expressed in *Escherichia coli*. *Nat Biotechnol* 25(5):563-565.
3. der Maur AA*, et al.* (2002) Direct *in vivo* screening of intrabody libraries constructed on a highly stable single-chain framework. *J Biol Chem* 277(47):45075-45085.
4. Jermutus L, Honegger A, Schwesinger F, Hanes J, & Pluckthun A (2001) Tailoring *in vitro* evolution for protein affinity or stability. *Proc Natl Acad Sci U S A* 98(1):75-80.
5. Koch H, Grafe N, Schiess R, & Pluckthun A (2006) Direct selection of antibodies from complex libraries with the protein fragment complementation assay. *J Mol Biol* 357(2):427-441.
6. Chen G, Dubrawsky I, Mendez P, Georgiou G, & Iverson BL (1999) *In vitro* scanning saturation mutagenesis of all the specificity determining residues in an antibody binding site. *Protein Eng* 12(4):349-356.
7. Jung ST*, et al.* (2010) Aglycosylated IgG variants expressed in bacteria that selectively bind FcgammaRI potentiate tumor cell killing by monocyte-dendritic cells. *Proc Natl Acad Sci U S A* 107(2):604-609.
